# An analysis of the Rayleigh–Stokes problem for a generalized second-grade fluid

**DOI:** 10.1007/s00211-014-0685-2

**Published:** 2014-11-26

**Authors:** Emilia Bazhlekova, Bangti Jin, Raytcho Lazarov, Zhi Zhou

**Affiliations:** 1 Institute of Mathematics and Informatics, Bulgarian Academy of Sciences, Acad. G. Bonchev str., Bl. 8, 1113 Sofia, Bulgaria; 2 Department of Computer Science, University College London, Gower Street, London, WC1E 6BT UK; 3 Department of Mathematics, Texas A&M University, College Station, TX 77843 USA

**Keywords:** 65M60, 65M15

## Abstract

We study the Rayleigh–Stokes problem for a generalized second-grade fluid which involves a Riemann–Liouville fractional derivative in time, and present an analysis of the problem in the continuous, space semidiscrete and fully discrete formulations. We establish the Sobolev regularity of the homogeneous problem for both smooth and nonsmooth initial data $v$, including $v\in L^2(\Omega )$. A space semidiscrete Galerkin scheme using continuous piecewise linear finite elements is developed, and optimal with respect to initial data regularity error estimates for the finite element approximations are derived. Further, two fully discrete schemes based on the backward Euler method and second-order backward difference method and the related convolution quadrature are developed, and optimal error estimates are derived for the fully discrete approximations for both smooth and nonsmooth initial data. Numerical results for one- and two-dimensional examples with smooth and nonsmooth initial data are presented to illustrate the efficiency of the method, and to verify the convergence theory.

## Introduction

In this paper, we study the homogeneous Rayleigh–Stokes problem for a generalized second-grade fluid with a fractional derivative model. Let $\Omega \subset {\mathbb {R}}^d (d=1,2,3)$ be a convex polyhedral domain with its boundary being $\partial \Omega$, and $T>0$ be a fixed time. Then the mathematical model is given by

1.1 $$\begin{aligned} \partial _t u-(1+\gamma \partial _t^\alpha )\Delta u&= f,\ \ \hbox {in }\Omega ,\ \ 0<t\le T;\nonumber \\ u&= 0,\ \ \hbox {on }\partial \Omega , \ 0<t\le T;\\ u(\cdot ,0)&= v,\ \ \hbox {in }\Omega ,\nonumber \end{aligned}$$

where $\gamma >0$ is a fixed constant, $v$ is the initial data, $\partial _t=\partial /\partial t$, and $\partial _t^\alpha$ is the Riemann–Liouville fractional derivative of order $\alpha \in (0,1)$ defined by [11, 24]:

$$\begin{aligned} \partial _t^\alpha f(t)=\frac{d}{d t}\int _0^t \omega _{1-\alpha }(t-s)f(s)\,ds, \qquad \omega _\alpha (t)=\frac{t^{\alpha -1}}{\Gamma (\alpha )}. \end{aligned}$$

The Rayleigh–Stokes problem (1.1) has received considerable attention in recent years. The fractional derivative $\partial _t^\alpha$ in the model is used to capture the viscoelastic behavior of the flow; see e.g. [5, 28] for derivation details. The model (1.1) plays an important role in describing the behavior of some non-Newtonian fluids.

In order to gain insights into the behavior of the solution of this model, there has been substantial interest in deriving a closed form solution for special cases; see, e.g. [5, 28, 32]. For example, Shen et al. [28] obtained the exact solution of the problem using the Fourier sine transform and fractional Laplace transform. Zhao and Yang [32] derived exact solutions using the eigenfunction expansion on a rectangular domain for the case of homogeneous initial and boundary conditions. The solutions obtained in these studies are formal in nature, and especially the regularity of the solution has not been studied. In Sect. 2 below, we fill this gap and establish the Sobolev regularity of the solution for both smooth and nonsmooth initial data. We would like to mention that Girault and Saadouni [7] analyzed the existence and uniqueness of a weak solution of a closely related time-dependent grade-two fluid model.

The exact solutions obtained in these studies involve infinite series and special functions, e.g., generalized Mittag–Leffler functions, and thus are inconvenient for numerical evaluation. Further, closed-form solutions are available only for a restricted class of problem settings. Hence, it is imperative to develop efficient and optimally accurate numerical algorithms for problem (1.1). This was considered earlier in [1, 2, 12, 21, 31]. Chen et al. [1] developed implicit and explicit schemes based on the finite difference method in space and the Grünwald–Letnikov discretization of the time fractional derivative, and analyzed their stability and convergence rates using the Fourier method. Of the same flavor is the work [2], where a scheme based on Fourier series expansion was considered. Wu [31] developed an implicit numerical approximation scheme by transforming problem (1.1) into an integral equation, and showed its stability and convergence by an energy argument. Lin and Jiang [12] described a method based on the reproducing kernel Hilbert space. Recently, Mohebbi et al. [21] compared a compact finite difference method with the radial basis function method. In all these studies, however, the error estimates were obtained under the assumption that the solution to (1.1) is sufficiently smooth and the domain $\Omega$ is a rectangle. Hence the interesting cases of nonsmooth data (the initial data or the right hand side) and general domains are not covered.

Theoretical studies on numerical methods for differential equations involving fractional derivatives have received considerable attention in the last decade. McLean and Mustapha [18, 22] analyzed piecewise constant and piecewise linear discontinuous Galerkin method in time, and derived error estimates for smooth initial data; see also [23] for related superconvergence results. In [8, 10], a space semidiscrete Galerkin finite element method (FEM) and lumped mass method for problem ${^C \partial _t^\alpha } u + A u = 0$ with $u(0)=v$ (with $A$ being an elliptic operator, and ${^C\partial _t^\alpha }$ being the Caputo derivative) has been analyzed. Almost optimal error estimates were established for initial data $v\in \dot{H}^{q}(\Omega ), -1\le q\le 2$, (see Sect. 2 below for the definition) by exploiting the properties of the two-parameter Mittag–Leffler function. Note that this includes weak (nonsmooth), $v \in L^2(\Omega )$, and very weak data, $v\in \dot{H}^{-1}(\Omega )$. In [19, Section 4], McLean and Thomée studied the following equation $\partial _t u + {\partial _t^{-\alpha }} Au = f$ (with ${\partial _t^{-\alpha }}$ being Riemann–Liouville integral and derivative operator for $\alpha \in (0,1)$ and $\alpha \in (-1,0)$, respectively), and derived $L^2(\Omega )$-error estimates for the space semidiscrete scheme for both $v\in L^2(\Omega )$ and $v\in \dot{H}^{2}(\Omega )$ (and suitably smooth $f$) and some fully discrete schemes based on Laplace transform were discussed. The corresponding $L^\infty (\Omega )$ estimates for data $v\in L^\infty (\Omega )$ and $Av\in L^\infty (\Omega )$ were derived in [20]. Lubich et al. [15] developed two fully discrete schemes for the problem $\partial _tu + \partial _t^{-\alpha } Au = f$ with $u(0)=v$ and $0<\alpha <1$ based on the convolution quadrature of the fractional derivative term, and derived optimal error estimates for nonsmooth initial data and right hand side. Cuesta et al. [4] considered the semi-linear counterpart of the model with convolution quadrature, which covers also the fractional diffusion case, i.e., $-1<\alpha <0$, and provided a unified framework for the error analysis with optimal error estimates in an abstract Banach space setting.

In this paper we develop a Galerkin FEM for problem (1.1) and derive optimal with respect to data regularity error estimates for both smooth and nonsmooth initial data. The approximation is based on the finite element space $X_h$ of continuous piecewise linear functions over a family of shape regular quasi-uniform partitions $\{\mathcal {T}_h\}_{0<h<1}$ of the domain $\Omega$ into $d$-simplexes, where $h$ is the maximum diameter. The semidiscrete Galerkin FEM for problem (1.1) is: find $u_h (t)\in X_h$ such that

1.2 $$\begin{aligned}&{(\partial _t u_{h},\chi )}+ \gamma \partial _t^\alpha a(u_h,\chi ) + a(u_h,\chi )= {(f, \chi )},\nonumber \\&\quad \forall \chi \in X_h,\ T \ge t >0, \quad u_h(0)=v_h, \end{aligned}$$

where $a(u,w)=(\nabla u, \nabla w) \text { for }\ u, \, w\in H_0^1(\Omega )$, and $v_h \in X_h$ is an approximation of the initial data $v$. Our default choices are the $L^2(\Omega )$ projection $v_h=P_hv$, assuming $v\in L^2(\Omega )$, and the Ritz projection $v_h=R_hv$, assuming $v\in \dot{H}^{2}(\Omega )$. Further, we develop two fully discrete schemes based on the backward Euler method and the second-order backward difference method and the related convolution quadrature for the fractional derivative term, which achieves respectively first and second-order accuracy in time. Error estimates optimal with respect to data regularity are provided for both semidiscrete and fully discrete schemes.

Our main contributions are as follows. First, in Theorem 2.1, using an operator approach from [25], we develop the theoretical foundations for our study by establishing the smoothing property and decay behavior of the solution to problem (1.1). Second, for both smooth initial data $v\in \dot{H}^{2}(\Omega )$ and nonsmooth initial data $v\in L^2(\Omega )$, we derive error estimates for the space semidiscrete scheme, cf. Theorems 3.1 and 3.2:

$$\begin{aligned}&\Vert u(t)-u_h(t)\Vert _{L^2(\Omega )} + h\Vert \nabla (u(t)-u_h(t))\Vert _{L^2(\Omega )} \\&\quad \le ch^2 t^{(q/2-1)(1-\alpha )}\Vert v\Vert _{\dot{H}^{q}(\Omega )},\quad q =0,2. \end{aligned}$$

The estimate for $v\in L^2(\Omega )$ deteriorates as $t$ approaches $0$. The error estimates are derived following an approach due to Fujita and Suzuki [6]. Next, in Theorems 4.1 and 4.2 we establish optimal $L^2(\Omega )$ error estimates for the two fully discrete schemes. The proof is inspired by the fundamental work of Cuesta et al. [4], which relies on known error estimates for convolution quadrature and bounds on the convolution kernel. We show for example, that the discrete solution $U_h^n$ by the backward Euler method (on a uniform grid in time with a time step size $\tau$) satisfies the following a priori error bound

$$\begin{aligned} \Vert U_h^n - u(t_n)\Vert _{L^2(\Omega )} \le c\left( \tau t_n^{-1 + (1-\alpha )q/2}+h^2 t_n^{(q/2-1)(1-\alpha )}\right) \Vert v\Vert _{\dot{H}^{q}(\Omega )},\quad q =0,2. \end{aligned}$$

A similar estimate holds for the second-order backward difference method.

The rest of the paper is organized as follows. In Sect. 2 we establish the Sobolev regularity of the solution. In Sect. 3, we analyze the space semidiscrete scheme, and derive optimal error estimates for both smooth and nonsmooth initial data. Then in Sect. 4, we develop two fully discrete schemes based on convolution quadrature approximation of the fractional derivative. Optimal error estimates are provided for both schemes. Finally in Sect. 5, numerical results for one- and two-dimensional examples are provided to illustrate the convergence theory. Throughout, the notation $c$ denotes a constant which may differ at different occurrences, but it is always independent of the solution $u$, mesh size $h$ and time step-size $\tau$.

## Regularity of the solution

In this section, we establish the Sobolev regularity of the solution to (1.1) in the homogeneous case $f\equiv 0$. We first recall preliminaries on the elliptic operator and function spaces. Then we derive the proper solution representation, show the existence of a weak solution, and establish the Sobolev regularity of the solution to the homogeneous problem. The main tool is the operator theoretic approach developed in [25]. Further, we give an alternative solution representation via eigenfunction expansion, and derive qualitative properties of the time-dependent components.

### Preliminaries

First we introduce some notation. For $q\ge -1$, we denote by $\dot{H}^{q}(\Omega ) \subset H^{-1}(\Omega )$ the Hilbert space induced by the norm

$$\begin{aligned} \Vert v\Vert _{\dot{H}^{q}(\Omega )}^2=\sum _{j=1}^\infty \lambda _j^q ( v,\varphi _j)^2, \end{aligned}$$

with $(\cdot ,\cdot )$ denoting the inner product in $L^2(\Omega )$ and $\{\lambda _j\}_{j=1}^\infty$ and $\{\varphi _j\}_{j=1}^\infty$ being respectively the Dirichlet eigenvalues and eigenfunctions of $-\Delta$ on the domain $\Omega$. As usual, we identify a function $f$ in $L^2(\Omega )$ with the functional $F$ in $H^{-1}(\Omega )\equiv (H_0^1(\Omega ))'$ defined by $\langle F,\phi \rangle = (f,\phi )$, for all $\phi \in H^1_0(\Omega )$. Then sets $\{\varphi _j\}_{j=1}^\infty$ and $\{\lambda _j^{1/2} \varphi _j\}_{j=1}^\infty$ form orthonormal basis in $L^2(\Omega )$ and $H^{-1}(\Omega )$, respectively. Thus $\Vert v \Vert _{\dot{H}^{-1}(\Omega )}=\Vert v \Vert _{H^{-1}(\Omega )}, \Vert v\Vert _{\dot{H}^{0}(\Omega )}=\Vert v\Vert _{L^2(\Omega )}=(v,v)^{1/2}$ is the norm in $L^2(\Omega ), \Vert v\Vert _{\dot{H}^{1}(\Omega )}$ is the norm in $H_0^1(\Omega )$ and $\Vert v\Vert _{\dot{H}^{2}(\Omega )}=\Vert \Delta v\Vert _{L^2(\Omega )}$ is equivalent to the norm in $H^2(\Omega )$ when $v=0$ on $\partial \Omega$ [29]. Note that $\dot{H}^s(\Omega ), s\ge -1$ form a Hilbert scale of interpolation spaces. Thus we denote $\Vert \cdot \Vert _{H_0^s(\Omega )}$ to be the norm on the interpolation scale between $H^1_0(\Omega )$ and $L^2(\Omega )$ for $s$ is in the interval $[0,1]$ and $\Vert \cdot \Vert _{H^{s}(\Omega )}$ to be the norm on the interpolation scale between $L^2(\Omega )$ and $H^{-1}(\Omega )$ when $s$ is in $[-1,0]$. Then, the $\dot{H}^s(\Omega )$ and $H_0^s(\Omega )$ norms are equivalent for any $s\in [0,1]$ by interpolation, and likewise the $\dot{H}^s(\Omega )$ and $H^{s}(\Omega )$ norms are equivalent for any $s\in [-1,0]$.

For $\delta >0$ and $\theta \in (0,\pi )$ we introduce the contour $\Gamma _{\delta ,\theta }$ defined by

$$\begin{aligned} \Gamma _{\delta ,\theta }=\left\{ re^{-{\mathrm {i}}\theta }:\ r\ge \delta \right\} \cup \left\{ \delta e^{i\psi }: \ |\psi |\le \theta \right\} \cup \left\{ re^{\mathrm {i}\theta }:\ r\ge \delta \right\} , \end{aligned}$$

where the circular arc is oriented counterclockwise, and the two rays are oriented with an increasing imaginary part. Further, we denote by $\Sigma _\theta$ the sector

$$\begin{aligned} \Sigma _\theta =\{z\in \mathbb {C};\ z\ne 0, |\arg z|<\theta \}. \end{aligned}$$

We recast problem (1.1) with $f\equiv 0$ into a Volterra integral equation by integrating both sides of the governing equation in (1.1)

2.1 $$\begin{aligned} u(x,t)=v(x)-\int _0^t k(t-s) A u(x,s)\,ds, \end{aligned}$$

where the kernel $k(t)$ is given by

$$\begin{aligned} k(t)=1+\gamma \omega _{1-\alpha }(t) \end{aligned}$$

and the operator $A$ is defined by $A=-\Delta$ with a domain $D(A)= H_0^1(\Omega ) \,\cap \, H^2(\Omega )$. The $H^2(\Omega )$ regularity of the elliptic problem is essential for our discussion, and it follows from the convexity assumption on the domain $\Omega$. It is well known that the operator $-A$ generates a bounded analytic semigroup of angle $\pi /2$, i.e., for any $\theta \in (0,\pi /2)$

2.2 $$\begin{aligned} \Vert (z+A)^{-1}\Vert \le M/|z|,\ \ \forall z\in \Sigma _{\pi -\theta }. \end{aligned}$$

Meanwhile, applying the Laplace transform to (2.1) yields

$$\begin{aligned} \widehat{u} (z) + \widehat{k}(z) A\widehat{u}(z) = z^{-1}v, \end{aligned}$$

i.e., $\widehat{u}(z) = H(z)v$, with the kernel $H(z)$ given by

2.3 $$\begin{aligned} H(z)=\frac{g(z)}{z}(g(z)I+A)^{-1},\quad g(z)=\frac{1}{\widehat{k}(z)}=\frac{z}{1+\gamma z^\alpha }, \end{aligned}$$

where $\widehat{k}$ is the Laplace transform of the function $k(t)$. Hence, by means of the inverse Laplace transform, we deduce that the solution operator $S(t)$ is given by

2.4 $$\begin{aligned} S(t)=\frac{1}{2\pi \mathrm {i}}\int _{\Gamma _{\delta ,\pi -\theta }} e^{z t} H(z)\, dz, \end{aligned}$$

where $$\delta >0, \theta \in (0,\pi /2)$$.

First we state one basic estimate about the kernel $g(z)=z/(1+\gamma z^\alpha )$.

#### **Lemma 2.1**

Fix $\theta \in (0,\pi )$, and let $g(z)$ be defined in (2.3). Then

2.5 $$\begin{aligned} g(z) \in \Sigma _{\pi -\theta }\quad and \quad |g(z)|\le c\min (|z|,|z|^{1-\alpha }),\quad \forall z\in \Sigma _{\pi -\theta }. \end{aligned}$$

#### *Proof*

Let $z \in \Sigma _{\pi -\theta }$, i.e. $z=r e^{\mathrm {i}\psi }, |\psi |<\pi -\theta , r>0$. Then by noting $\alpha \in (0,1)$,

2.6 $$\begin{aligned} g(z) = \frac{re^{\mathrm {i}\psi }}{1+\gamma r^\alpha e^{\alpha \mathrm {i}\psi }} = \frac{r e^{\mathrm {i}\psi }+\gamma r^{\alpha +1}e^{\mathrm {i}(1-\alpha )\psi }}{(1+\gamma r^{\alpha } \cos (\alpha \psi ))^2+(\gamma r^\alpha \sin (\alpha \psi ))^2}\in \Sigma _{\pi -\theta }. \end{aligned}$$

To prove (2.5) we note that

2.7 $$\begin{aligned} |1+\gamma z^\alpha |^2=1+2\gamma r^\alpha \cos (\alpha \psi )+\gamma ^2 r^{2\alpha } >1+2\gamma r^\alpha \cos (\alpha \pi )+\gamma ^2 r^{2\alpha }. \end{aligned}$$

Let $b=\cos (\alpha \pi )$. Since the function $f(x)=1+2bx+x^2$ attains its minimum at $x=-b$, with a minimum value $f_{min}=f(-b)=1-b^2$, it follows from (2.7) that

$$\begin{aligned} |1+\gamma z^\alpha |^2>1-\cos ^2(\alpha \pi )=\sin ^2(\alpha \pi ). \end{aligned}$$

Since $\sin (\alpha \pi )>0$, this leads to the first assertion

$$\begin{aligned} |g(z)|=\left| \frac{z}{1+\gamma z^\alpha } \right| <\frac{1}{\sin (\alpha \pi )}|z|. \end{aligned}$$

From (2.7) it follows that

2.8 $$\begin{aligned} |1+\gamma z^\alpha |^2>(1+\gamma r^{\alpha } \cos (\alpha \pi ))^2+(\gamma r^\alpha \sin (\alpha \pi ))^2\ge \sin ^2(\alpha \pi )\gamma ^2r^{2\alpha }, \end{aligned}$$

and consequently, we get

$$\begin{aligned} |g(z)| = \bigg |\frac{z}{1+\gamma z^\alpha }\bigg | \le \frac{r}{\gamma r^\alpha \sin (\alpha \pi )}=\frac{1}{\gamma \sin (\alpha \pi )}|z|^{1-\alpha }. \end{aligned}$$

This completes the proof of the lemma.$\square$

### A priori estimates of the solution

Now we can state the regularity to problem (1.1) with $f\equiv 0$.

#### **Theorem 2.1**

For any $v\in L^2(\Omega )$ and $f\equiv 0$ there exists a unique solution $u$ to problem (1.1) and

$$\begin{aligned} u=S(t)v\in C([0,T];L^2(\Omega ))\cap C((0,T];H^2(\Omega )\cap H_0^1(\Omega )). \end{aligned}$$

Moreover, the following stability estimates hold for $t\in (0,T]$ and $\nu =0,1$:

2.9 $$\begin{aligned}&\Vert A^\nu S^{(m)}(t)v\Vert _{L^2(\Omega )}\le c t^{-m-\nu (1-\alpha )}\Vert v\Vert _{L^2(\Omega )},\ v\in L^2(\Omega ), m\ge 0,\end{aligned}$$

2.10 $$\begin{aligned}&\Vert A^\nu S^{(m)}(t)v\Vert _{L^2(\Omega )}\le c_T t^{-m+(1-\nu )(1-\alpha )}\Vert Av\Vert _{L^2(\Omega )},\ v\in D(A), \nu +m\ge 1,\nonumber \\ \end{aligned}$$

where $c,c_T>0$ are constants depending on $d, \Omega , \alpha , \gamma , M$ and $m$, and the constant $c_T$ also depends on $T$.

#### *Proof*

By Lemma 2.1 and (2.2) we obtain

2.11 $$\begin{aligned} \Vert (g(z)I+A)^{-1}\Vert \le M/|g(z)|,\ \ z\in \Sigma _{\pi -\theta }, \end{aligned}$$

and we deduce from (2.3) and (2.11) that

2.12 $$\begin{aligned} \Vert H(z)\Vert \le {M}/{|z|},\ \ z\in \Sigma _{\pi -\theta }. \end{aligned}$$

Then by [25, Theorem 2.1 and Corollary 2.4], for any $v\in L^2(\Omega )$ there exists a unique solution $u$ of (2.1) and it is given by

$$\begin{aligned} u(t)=S(t)v. \end{aligned}$$

It remains to show the estimates.

Let $t>0, \theta \in (0,\pi /2), \delta >0$. We choose $\delta =1/t$ and denote for short

2.13 $$\begin{aligned} \Gamma =\Gamma _{1/t,\pi -\theta }. \end{aligned}$$

First we derive (2.9) for $\nu =0$ and $m\ge 0$. From (2.4) and (2.12) we deduce

$$\begin{aligned} \Vert S^{(m)}(t)\Vert&= \left\| \frac{1}{2\pi \mathrm {i}} \int _{\Gamma } z^m e^{zt} H(z)\, dz \right\| \le c\int _{\Gamma } |z|^m e^{\mathfrak {R}(z)t} \Vert H(z)\Vert \, |dz|\\&\le c\left( \int _{1/t}^\infty r^{m-1}e^{-rt\cos \theta } \,dr + \int _{-\pi +\theta }^{\pi -\theta }e^{\cos \psi }t^{-m}\,d\psi \right) \le ct^{-m}. \end{aligned}$$

Next we prove estimate (2.9) for $\nu =1$ and $m\ge 0$. By applying the operator $A$ to both sides of (2.4) and differentiating we arrive at

2.14 $$\begin{aligned} AS^{(m)}(t)=\frac{1}{2\pi \mathrm {i}}\int _\Gamma z^m e^{z t} AH(z) dz. \end{aligned}$$

Using the identity

$$\begin{aligned} AH(z)=\left( -H(z)+z^{-1}I\right) g(z), \end{aligned}$$

it follows from (2.12) and Lemma 2.1 that

2.15 $$\begin{aligned} \left\| AH(z)\right\| \le (M+1)|z^{-1}g(z)|\le c\min (1,|z|^{-\alpha }),\quad z\in \Sigma _{\pi -\theta }. \end{aligned}$$

By taking $\left\| AH(z)\right\| \le c|z|^{-\alpha }$, we obtain from (2.14)

$$\begin{aligned} \Vert AS^{(m)}(t)\Vert&\le c\int _{\Gamma } |z|^{m-\alpha } e^{\mathfrak {R}(z)t} \, |dz| \\&\le c\left( \int _{1/t}^\infty r^{m-\alpha }e^{-rt\cos \theta } \,dr + \int _{-\pi +\theta }^{\pi -\theta }e^{\cos \psi }t^{-m-1+\alpha }\,d\psi \right) \le c t^{-m-1+\alpha }. \end{aligned}$$

This shows estimate (2.9). To prove estimate (2.10) with $\nu =0$ we observe that

$$\begin{aligned} S^{(m)}(t)v&= \frac{1}{2\pi \mathrm {i}}\int _{\Gamma } z^m e^{zt} \frac{g(z)}{z} (g(z)I+A)^{-1} v \,dz \\&= \frac{1}{2\pi \mathrm {i}}\int _{\Gamma } z^{m-1}e^{zt} g(z) A^{-1}(g(z)I+A)^{-1} Av dz. \end{aligned}$$

Now by noting the identity

$$\begin{aligned} g(z)A^{-1}(g(z)I+A)^{-1}= A^{-1}-(g(z)I+A)^{-1} \end{aligned}$$

and the fact that $\int _{\Gamma } z^{m-1}e^{zt}\,dz=0$ for $m\ge 1$, we have

$$\begin{aligned} S^{(m)}(t)v&= \frac{1}{2\pi \mathrm {i}}\int _{\Gamma }z^{m-1} e^{zt} v\, dz - \frac{1}{2\pi \mathrm { i}}\int _{\Gamma } z^{m-1}e^{zt}(g(z)I+A)^{-1} \,dzA v\\&= -\frac{1}{2\pi \mathrm {i}}\int _{\Gamma } z^{m-1}e^{zt}(g(z)I+A)^{-1} \,dzA v.\\ \end{aligned}$$

By (2.11) we obtain

$$\begin{aligned} \Vert (g(z)I+A)^{-1}\Vert \le M |g(z)|^{-1}=M\bigg |\frac{1+\gamma z^\alpha }{z}\bigg | \le M(|z|^{-1}+\gamma |z|^{\alpha -1}), \end{aligned}$$

and thus using this estimate, we get

$$\begin{aligned} \Vert S^{(m)}(t)v\Vert _{L^2(\Omega )}&\le c\left( \int _{\Gamma } |z|^{m-1}e^{\mathfrak {R}(z)t}\Vert (g(z)I+A)^{-1}\Vert \,|dz|\right) \Vert Av \Vert _{L^2(\Omega )}\\&\le c\left( \int _{1/t}^\infty e^{-rt\cos \theta }(r^{m-2}+\gamma r^{m+\alpha -2}) \,dr\right. \\&\left. + \int _{-\pi +\theta }^{\pi -\theta } e^{\cos \psi } (t^{-m+1}+\gamma t^{-m+1-\alpha }) \,d\psi \right) \Vert Av \Vert _{L^2(\Omega )}\\&\le c(t^{-m+1}+\gamma t^{-m+1-\alpha })\Vert Av \Vert _{L^2(\Omega )}. \end{aligned}$$

Since $t^{-m+1}\le T^{\alpha }t^{-m+1-\alpha }$ for $t\in (0,T]$, we deduce

$$\begin{aligned} \Vert S^{(m)}(t)v\Vert _{L^2(\Omega )} \le c_T t^{-m+1-\alpha }\Vert Av \Vert _{L^2(\Omega )}, \ \ t\in (0,T], \end{aligned}$$

with $c_T=c(T^\alpha +\gamma )$. Lastly, note that (2.10) with $\nu =1$ is equivalent to (2.9) with $\nu =0$ and $v$ replaced by $Av$.$\square$

#### *Remark 2.1*

We note that this argument is applicable to any sectorial operator $A$, including the Riemann–Liouville fractional derivative operator in space [9].

Further, the estimates in Theorem 2.1 imply the following result by interpolation.

#### *Remark 2.2*

The solution $S(t)v$ to problem (1.1) with $f\equiv 0$ satisfies

$$\begin{aligned} \Vert S^{(m)}(t)v\Vert _{\dot{H}^{p}(\Omega )} \le ct^{-m-(1-\alpha )(p-q)/2}\Vert v \Vert _{\dot{H}^{q}(\Omega )}\quad \forall t\in (0,T], \end{aligned}$$

where for $m=0$ and $0\le q\le p \le 2$ or $m>0$ and $0\le p, \ q\le 2$.

### Further discussions on the behavior of the solution

The estimate (2.9) holds for any $t>0$. However, in the case $\nu =1$ and $m=0$ we can improve this estimate for large $t>0$. Namely, if we apply the bound $\Vert AH(z)\Vert \le M$ from (2.15) in the estimate of (2.14), we get the following sharper bound for large $t$:

#### *Remark 2.3*

For $v\in L^2(\Omega )$ we have the following bound

2.16 $$\begin{aligned} \Vert A S(t)v\Vert _{L^2(\Omega )}\le c t^{-1}\Vert v\Vert _{L^2(\Omega )},\ \ t>0, \end{aligned}$$

which is sharper than (2.9) for large $t$. This bound together with (2.9) with $\nu =0, m=1$, imply the following a priori estimate for the solution of problem (1.1):

$$\begin{aligned} \Vert \partial _t u\Vert _{L^2(\Omega )}+\Vert u\Vert _{\dot{H}^{2}(\Omega )}+\Vert \partial _t^\alpha u\Vert _{\dot{H}^{2}(\Omega )}\le ct^{-1}\Vert v\Vert _{L^2(\Omega )} \quad { for} \ { large}\ t>0. \end{aligned}$$

Further, by applying eigenfunction expansion, the solution of the Rayleigh–Stokes problem (1.1) can be written in the form

$$\begin{aligned} u(x,t)=\sum _{j=1}^\infty (v,\varphi _j) u_j(t)\varphi _j(x)+\sum _{j=1}^\infty \left( \int _0^t u_j(t-\tau )f_j(\tau )\,d\tau \right) \varphi _j(x), \end{aligned}$$

where $f_j(t)=( f(.,t),\varphi _j)$ and $u_j(t)$ satisfies the following equation:

2.17 $$\begin{aligned} u'_j(t)+\lambda _j(1+\gamma \partial _t^\alpha )u_j(t)=0, \ \ u_j(0)=1. \end{aligned}$$

To solve (2.17) we apply Laplace transform and use the identities

2.18 $$\begin{aligned}&{\mathcal {L}}\{u'\}(z)=z {\mathcal {L}}\{ u\}(z)-u(0)\end{aligned}$$

2.19 $$\begin{aligned}&{\mathcal {L}}\{\partial _t^\alpha u\}(z)=z^\alpha {\mathcal {L}}\{ u\}(z),\ \ \alpha \in (0,1), \end{aligned}$$

which hold for functions $u(t)$, continuous for $t>0$, and such that $u(0)$ is finite [16, equation (1.15)]. In this way, for the Laplace transform of $u_j(t)$, one arrives at

$$\begin{aligned} {\mathcal {L}} \{u_j\}(z)=\frac{1}{z+\gamma \lambda _j z^\alpha +\lambda _j}. \end{aligned}$$

Based on this representation, in the next theorem we summarize some properties of the time-dependent components $u_j(t)$, which are useful in the study of the solution behavior, including the inhomogeneous problem.

Recall that a function $u(t)$ is said to be completely monotone if and only if

$$\begin{aligned} (-1)^n u^{(n)}(t)\ge 0, \hbox {\ for\ all\ }t\ge 0, \quad n=0,1,\ldots \end{aligned}$$

#### **Theorem 2.2**

The functions $u_j(t),\ j=1,2,\ldots ,$ have the following properties:

$$\begin{aligned}&u_j(0)=1,\ 0<u_j(t)\le 1, \ t\ge 0,\\&u_j(t)\ { are}\ { completely}\ { monotone}\ { for}\ t\ge 0, \\&|\lambda _j u_j(t)|\le c\min \{t^{-1},t^{\alpha -1}\},\ \ t> 0, \ \\&\int _0^T |u_j(t)|\,dt<\frac{1}{\lambda _j},\ \ T>0. \end{aligned}$$

where the constant $c$ does not depend on $j$ and $t$.

#### *Proof*

We introduce the auxiliary functions $v_j(t)$ defined by their Laplace transforms

2.20 $$\begin{aligned} \mathcal {L} \{v_j\}(z)=\frac{1+\gamma \lambda _j z^{\alpha -1}}{z+\gamma \lambda _j z^\alpha +\lambda _j}. \end{aligned}$$

By the property of the Laplace transform $u(0)=\lim _{z\rightarrow +\infty } z \widehat{u}(z)$ we obtain $u_j(0)=1$ and $v_j(0)=1$. Further, taking the inverse Laplace transform of (2.17), we get

$$\begin{aligned} u_j(t)=\frac{1}{2\pi \mathrm {i}} \int _{Br}e^{zt}\frac{1}{z+\gamma \lambda _j z^\alpha +\lambda _j}\,dz, \end{aligned}$$

where $Br=\{z;\ \mathfrak {R}z=\sigma ,\ \sigma >0\}$ is the Bromwich path [30]. The function under the integral has a branch point $0$, so we cut off the negative part of the real axis. Note that the function $z+\gamma \lambda _j z^\alpha +\lambda _j$ has no zero in the main sheet of the Riemann surface including its boundaries on the cut. Indeed, if $z=\varrho e^{i\theta }$, with $\varrho >0, \theta \in (-\pi ,\pi )$, then

$$\begin{aligned} \mathfrak {I}\{z+\gamma \lambda _j z^\alpha +\lambda _j\}=\varrho \sin \theta +\gamma \lambda _j\varrho ^\alpha \sin \alpha \theta \ne 0,\quad \theta \ne 0, \end{aligned}$$

since $\sin \theta$ and $\sin \alpha \theta$ have the same sign and $\lambda _j, \gamma >0$. Hence, $u_j(t)$ can be found by bending the Bromwich path into the Hankel path $Ha(\varepsilon )$, which starts from $-\infty$ along the lower side of the negative real axis, encircles the disc $|z|=\varepsilon$ counterclockwise and ends at $-\infty$ along the upper side of the negative real axis. By taking $\varepsilon \rightarrow 0$ we obtain

$$\begin{aligned} u_j(t)= \int _0^\infty e^{-rt}K_j(r)\,dr, \end{aligned}$$

where

$$\begin{aligned} K_j(r)=\frac{\gamma }{\pi } \frac{\lambda _j r^{\alpha } \sin \alpha \pi }{(-r+\lambda _j \gamma r^\alpha \cos \alpha \pi +\lambda _j)^2+(\lambda _j \gamma r^\alpha \sin \alpha \pi )^2}. \end{aligned}$$

Since $\alpha \in (0,1)$, and $\lambda _j,\gamma >0$, there holds $K_j(r)>0$ for all $r>0$. Hence, by Bernstein’s theorem, $u_j(t)$ are completely monotone functions. In particular, they are positive and monotonically decreasing. This shows the first two assertions.

In the same way we prove that the functions $v_j(t)$ are completely monotone and hence $0<v_j(t)\le 1$. By (2.18), and (2.20),

$$\begin{aligned} {\mathcal {L}}\{v_j'\}(z) =z{\mathcal {L}}\{v_j\}(z)-v_j(0)=z{\mathcal {L}}\{v_j\}(z)-1=-\lambda _j{\mathcal {L}}\{u_j\}(z), \end{aligned}$$

which, upon taking the inverse Laplace transform, implies $u_j(t)=- v_j'(t)/\lambda _j$. Now the third assertion follows by

$$\begin{aligned} \int _0^T |u_j(t)|\,dt =\int _0^T u_j(t)\,dt=-\frac{1}{\lambda _j}\int _0^T v'_j(t)\,dt=\frac{1}{\lambda _j}(1-v_j(T))<\frac{1}{\lambda _j}. \end{aligned}$$

Last, using the representation

$$\begin{aligned} u_j(t)=\frac{1}{2\pi \mathrm {i}} \int _{\Gamma }e^{zt}\frac{1}{z+\gamma \lambda _j z^\alpha +\lambda _j}\,dz=\frac{1}{2\pi \mathrm {i}} \int _{\Gamma }e^{zt}H(z,\lambda _j)\,dz \end{aligned}$$

with

$$\begin{aligned} H(z,\lambda _j)=\frac{g(z)}{z}(g(z)+\lambda _j)^{-1}, \end{aligned}$$

where the function $g(z)$ is defined as in (2.3), the last assertion follows by applying the argument from the proof of Theorem 2.1 with $A$ replaced by $\lambda _j>0$ and using the following estimate analogous to (2.15):

$$\begin{aligned} |\lambda _jH(z,\lambda _j)|\le M\min (1,|z|^{-\alpha }),\quad z\in \Sigma _{\pi -\theta }. \end{aligned}$$

This completes the proof of the proposition.$\square$

By Theorem 2.1, for any $\alpha \in (0,1)$, the solution operator $S$ has a smoothing property in space of order two. In the limiting case $\alpha =1$, however, it does not have any smoothing property. To see this, we consider the eigenfunction expansion:

2.21 $$\begin{aligned} u(x,t) = S(t)v = \sum _{j=1}^\infty (v,\varphi _j)u_j(t)\varphi _j(x). \end{aligned}$$

In the case $\alpha =1$ we deduce from (2.17) and (2.18)

$$\begin{aligned} \mathcal {L} \{u_j\}(z)=\frac{1+\gamma \lambda _j}{z+\gamma \lambda _jz+\lambda _j}, \quad \hbox {which implies} \quad u_j(t)=e^{-\frac{\lambda _j}{1+\gamma \lambda _j}t}. \end{aligned}$$

This shows that the problem does not have smoothing property.

#### *Remark 2.4*

We observe that if $v\in L^2(\Omega )$, then $\Vert u(t) \Vert _{\dot{H}^2(\Omega )}$ behaves like $t^{\alpha -1}$ as $t\rightarrow 0$. This behavior is the identical with that of the solution to the subdiffusion equation; see [17, Theorem 4.1] and [26, Theorem 2.1]. However, as $t\rightarrow \infty , \Vert u(t) \Vert _{\dot{H}^2(\Omega )}$ decays like $t^{-1}$, as in the case of standard diffusion equation. The solution $u(t)$ of (1.1) decays like $t^{-1}$ for $t\rightarrow \infty$. This is faster than $t^{\alpha -1}$, the decay of the solution to subdiffusion equation [26, Corollary 2.6], but much slower than the exponential decay for the diffusion equation.

We may extend Theorem 2.1 to the case of very weak initial data, i.e., $v\in \dot{H}^{q}(\Omega )$ with $-1<q<0$. Obviously, for any $t > 0$ the function $u(t) = S(t)v$ satisfies Eq. (1.1) in the sense of $\dot{H}^{q}(\Omega )$. Then we appeal to the expansion (2.21). Repeating the argument of Theorem 2.1 yields $\Vert S(t)v-v \Vert _{\dot{H}^{q}(\Omega )} \le c \Vert v \Vert _{\dot{H}^{q}(\Omega )}.$ By Lebesgue’s dominated convergence theorem we deduce

$$\begin{aligned} \lim _{t \rightarrow 0^+} \Vert S(t)-v \Vert _{\dot{H}^{q}(\Omega )}^2 = \lim _{t \rightarrow 0^+} \sum _{j=1}^\infty \lambda _j^q (u_j(t)-1)^2(v,\varphi _j)^2=0. \end{aligned}$$

Hence, the function $u(t) = S(t)v$ satisfies (1.1) and for $t\rightarrow 0$ converges to $v$ in $\dot{H}^{q}(\Omega )$, i.e., $u(t)=S(t)v$ does represent a solution. Further, the argument of Theorem 2.1 yields $u(t)=S(t)v \in \dot{H}^{2+q}(\Omega )$ for any $t>0$.

## Semidiscrete Galerkin finite element method

In this section we consider the space semidiscrete finite element approximation and derive optimal error estimates for the homogeneous problem.

### Semidiscrete Galerkin scheme

First we recall the $L^2(\Omega )$-orthogonal projection $P_h:L^2(\Omega )\rightarrow X_h$ and the Ritz projection $R_h:H^1_0(\Omega )\rightarrow X_h$, respectively, defined by

$$\begin{aligned} (P_h \varphi ,\chi )&= (\varphi ,\chi ) \quad \forall \chi \in X_h,\\ (\nabla R_h \varphi ,\nabla \chi )&= (\nabla \varphi ,\nabla \chi ) \quad \forall \chi \in X_h. \end{aligned}$$

For $\varphi \in \dot{H}^{-s}(\Omega )$ for $0< s \le 1$, the $L^2(\Omega )$-projection $P_h$ is not well-defined. Nonetheless, one may view $(\varphi ,\chi )$ for $\chi \in X_h \subset \dot{H}^{s}$ as the duality pairing between the spaces $\dot{H}^{s}(\Omega )$ and $\dot{H}^{-s}(\Omega )$ and define $P_h$ in the same manner.

The Ritz projection $R_h$ and the $L^2$-projection $P_h$ have the following properties.

#### **Lemma 3.1**

Let the mesh $X_h$ be quasi-uniform. Then the operators $R_h$ and $P_h$ satisfy:

$$\begin{aligned}&\Vert R_h \varphi -\varphi \Vert _{L^2(\Omega )}+h\Vert \nabla (R_h \varphi -\varphi )\Vert _{L^2(\Omega )}\le ch^q\Vert \varphi \Vert _{\dot{H}^q(\Omega )}\quad \forall \varphi \in \dot{H}^q(\Omega ), \ q=1,2,\\&\Vert P_h \varphi -\varphi \Vert _{L^2(\Omega )}+h\Vert \nabla (P_h \varphi -\varphi )\Vert _{L^2(\Omega )}\le ch^q\Vert \varphi \Vert _{\dot{H}^q(\Omega )}\quad \forall \varphi \in \dot{H}^q(\Omega ), \ q=1,2. \end{aligned}$$

In addition, $P_h$ is stable on $\dot{H}^{q}(\Omega )$ for $-1\le q \le 1$.

Upon introducing the discrete Laplacian $\Delta _h: X_h\rightarrow X_h$ defined by

3.1 $$\begin{aligned} -(\Delta _h\varphi ,\chi )=(\nabla \varphi ,\nabla \chi )\quad \forall \varphi ,\,\chi \in X_h, \end{aligned}$$

and $f_h= P_h f$, we may write the spatially discrete problem (1.2) as to find $u_h \in X_h$ such that

3.2 $$\begin{aligned} \partial _t u_h - (1 +\gamma \partial _t^\alpha )\Delta _h u_h = f_h, \quad u_h(0)=v_h, \end{aligned}$$

where $v_h\in X_h$ is a suitable approximation to the initial condition $v$. Accordingly, the solution operator $S_h(t)$ for the semidiscrete problem (1.2) is given by

3.3 $$\begin{aligned} S_h(t)=\frac{1}{2\pi \mathrm {i}}\int _\Gamma e^{z t} H_h(z) \, dz \quad \hbox {with} \quad H_h(z)=\frac{g(z)}{z}(g(z)I+A_h)^{-1}, \end{aligned}$$

where $\Gamma$ is the contour defined in (2.13) and $A_h=-\Delta _h$. Further, with the eigenpairs $\{(\lambda _j^h,\varphi _j^h)\}$ of the discrete Laplacian $-\Delta _h$, we define the discrete norm $|||\cdot |||_{\dot{H}^p(\Omega )}$ on the space $X_h$ for any $p\in \mathbb {R}$

$$\begin{aligned} |||{\varphi }|||_{\dot{H}^p(\Omega )}^2 = \sum _{j=1}^N(\lambda _j^h)^p(\varphi ,\varphi _j^h)^2\quad \forall \varphi \in X_h. \end{aligned}$$

The stability of the operator $S_h(t)$ is given below. The proof is similar to that of Theorem 2.1, and hence omitted.

#### **Lemma 3.2**

Let $S_h(t)$ be defined by (3.2) and $v_h \in X_h$. Then

$$\begin{aligned} |||S_h^{(m)}(t)v_h |||_{\dot{H}^p(\Omega )} \le ct^{-m-(1-\alpha )(p-q)/2}|||v_h |||_{\dot{H}^q(\Omega )}, \quad \forall 0<t\le T, \end{aligned}$$

where for $m=0$ and $0\le q\le p \le 2$ or $m>0$ and $0\le p , ~~q\le 2$.

Now we derive error estimates for the semidiscrete Galerkin scheme (3.2) using an operator trick, following the interesting work of Fujita and Suzuki [6]. We note that similar estimates follow also from the technique in [10], but at the expense of an additional logarithmic factor $|\ln h|$ in the case of nonsmooth initial data.

The following lemma plays a key role in deriving error estimates.

#### **Lemma 3.3**

For any $\varphi \in H_0^1(\Omega )$ and $z\in \Sigma _{\pi -\theta }=\left\{ z: |\arg (z)|\le \pi -\theta \right\}$ for $\theta \in (0,\pi /2)$, there holds

3.4 $$\begin{aligned} |g(z)| \Vert \varphi \Vert _{L^2(\Omega )}^2 + \Vert \nabla \varphi \Vert _{L^2(\Omega )}^2 \le c\left| g(z)\Vert \varphi \Vert _{L^2(\Omega )}^2 + (\nabla \varphi ,\nabla \varphi )\right| . \end{aligned}$$

#### *Proof*

By [6, Lemma 7.1], we have that for any $z\in \Sigma _{\pi -\theta }$

$$\begin{aligned} |z| \Vert \varphi \Vert _{L^2(\Omega )}^2 + \Vert \nabla \varphi \Vert _{L^2(\Omega )}^2 \le c\left| z\Vert \varphi \Vert _{L^2(\Omega )}^2 + (\nabla \varphi ,\nabla \varphi )\right| . \end{aligned}$$

Alternatively, it follows from the inequality

$$\begin{aligned} \gamma |z| + \beta \le \frac{|\gamma z+\beta |}{\sin \frac{\theta }{2}}\quad \hbox {for } \gamma ,\beta \ge 0, z\in \Sigma _{\pi -\theta }, \end{aligned}$$

with the choice $\gamma =\Vert \varphi \Vert _{L^2(\Omega )}^2$ and $\beta =\Vert \nabla \varphi \Vert _{L^2(\Omega )}^2=(\nabla \varphi ,\nabla \varphi )$. By Lemma 2.1, $g(z)\in \Sigma _{\pi -\theta }$ for all $z\in \Sigma _{\pi -\theta }$, and this completes the proof.$\square$

The next lemma shows an error estimate between $(g(z)I+A)^{-1}v$ and its discrete analogue $(g(z)I+A_h)^{-1}P_h v$.

#### **Lemma 3.4**

Let $v\in L^2(\Omega ) , z\in \Sigma _{\pi -\theta }, w=(g(z)I+A)^{-1}v$, and $w_h=(g(z)I+A_h)^{-1}P_h v$. Then there holds

3.5 $$\begin{aligned} \Vert w_h-w \Vert _{L^2(\Omega )} + h\Vert \nabla (w_h-w)\Vert _{L^2(\Omega )} \le ch^2 \Vert v \Vert _{L^2(\Omega )}. \end{aligned}$$

#### *Proof*

By the definition, $w$ and $w_h$ respectively satisfy

$$\begin{aligned} g(z)(w,\chi )+(\nabla w,\nabla \chi )&= (v,\chi ),\quad \forall \chi \in H^{1}_{0}(\Omega ),\\ g(z)(w_h,\chi )+(\nabla w,\nabla \chi )&= (v,\chi ),\quad \forall \chi \in X_h. \end{aligned}$$

Subtracting these two identities yields the following orthogonality relation for the error $e=w-w_h$:

3.6 $$\begin{aligned} g(z)(e,\chi ) + (\nabla e, \nabla \chi ) = 0, \quad \forall \chi \in X_h. \end{aligned}$$

This and Lemma 3.3 imply that for any $\chi \in X_h$

$$\begin{aligned} |g(z)| \Vert e\Vert _{L^2(\Omega )}^2 + \Vert \nabla e \Vert _{L^2(\Omega )}^2&\le c \left| g(z)\Vert e \Vert _{L^2(\Omega )}^2 + (\nabla e, \nabla e)\right| \\&= c \left| g(z)(e,w-\chi ) + (\nabla e, \nabla (w-\chi ))\right| . \end{aligned}$$

By taking $\chi =\pi _h w$, the Lagrange interpolant of $w$, and using the Cauchy–Schwarz inequality, we arrive at

3.7 $$\begin{aligned}&|g(z)| \Vert e \Vert _{L^2(\Omega )}^2 + \Vert \nabla e \Vert _{L^2(\Omega )}^2 \nonumber \\&\quad \le c \left( |g(z)| h\Vert e\Vert _{L^2(\Omega )} \Vert \nabla w\Vert _{L^2(\Omega )}+ h \Vert \nabla e\Vert _{L^2(\Omega )} \Vert w \Vert _{\dot{H}^2 (\Omega )} \right) . \end{aligned}$$

Appealing again to Lemma 3.3 with the choice $\varphi =w$, we obtain

$$\begin{aligned} |g(z)| \Vert w \Vert _{L^2(\Omega )}^2 + \Vert \nabla w\Vert _{L^2(\Omega )}^2 \le c|((g(z)I+A)w,w)|\le c\Vert v \Vert _{L^2(\Omega )}\Vert w\Vert _{L^2(\Omega )}. \end{aligned}$$

Consequently

3.8 $$\begin{aligned} \Vert w \Vert _{L^2(\Omega )} \le c|g(z)|^{-1}\Vert v \Vert _{L^2(\Omega )}\quad \hbox {and}\quad \Vert \nabla w \Vert _{L^2(\Omega )} \le c|g(z)|^{-1/2}\Vert v \Vert _{L^2(\Omega )}. \end{aligned}$$

In view of (3.8), a bound on $\Vert w \Vert _{\dot{H}^{2}(\Omega )}$ can be derived

$$\begin{aligned} \Vert w \Vert _{\dot{H}^{2}(\Omega )}&= \Vert A w \Vert _{L^2(\Omega )} = c\Vert (-g(z)I+g(z)I+A)(g(z)I+A)^{-1}v \Vert _{L^2(\Omega )}\\&\le c\left( \Vert v \Vert _{L^2(\Omega )}+|g(z)|\Vert w\Vert _{L^2(\Omega )}\right) \le c\Vert v\Vert _{L^2(\Omega )}. \end{aligned}$$

It follows from this and (3.7) that

$$\begin{aligned} |g(z)| \Vert e\Vert _{L^2(\Omega )}^2 + \Vert \nabla e\Vert _{L^2(\Omega )}^2 \le ch \Vert v \Vert _{L^2(\Omega )}\left( |g(z)|^{1/2}\Vert e\Vert _{L^2(\Omega )} + \Vert \nabla e\Vert _{L^2(\Omega )} \right) , \end{aligned}$$

and this yields

3.9 $$\begin{aligned} |g(z)| \Vert e\Vert _{L^2(\Omega )}^2 + \Vert \nabla e\Vert _{L^2(\Omega )}^2\le ch^{2}\Vert v \Vert _{L^2(\Omega )}^2. \end{aligned}$$

This gives the desired bound on $\Vert \nabla e\Vert _{L^2(\Omega )}$. Next, we derive the estimate on $\Vert e\Vert _{L^2(\Omega )}$ by a duality argument. For $\varphi \in L^2(\Omega )$, by setting

$$\begin{aligned} \psi =(g(z)I+A)^{-1}\varphi \quad \text {and}\quad \psi _h=(g(z)I+A_h)^{-1}P_h\varphi \end{aligned}$$

we have by duality

$$\begin{aligned} \Vert e \Vert _{L^2(\Omega )} \le \sup _{\varphi \in L^2(\Omega )}\frac{|(e,\varphi )|}{\Vert \varphi \Vert _{L^2(\Omega )}} =\sup _{\varphi \in L^2(\Omega )}\frac{|g(z)(e,\psi )+(\nabla e,\nabla \psi )|}{\Vert \varphi \Vert _{L^2(\Omega )}}. \end{aligned}$$

Then the desired estimate follows from (3.6) and (3.9) by

$$\begin{aligned} |g(z)(e,\psi )+(\nabla e,\nabla \psi )|&= |g(z)(e,\psi -\psi _h)+(\nabla e,\nabla (\psi -\psi _h))|\\&\le |g(z)|^{1/2}\Vert e\Vert _{L^2(\Omega )} |g(z)|^{1/2}\Vert \psi -\psi _h \Vert _{L^2(\Omega )}\\&+ \Vert \nabla e\Vert _{L^2(\Omega )}\Vert \nabla (\psi -\psi _h) \Vert _{L^2(\Omega )}\\&\le ch^{2} \Vert v \Vert _{L^2(\Omega )}\Vert \varphi \Vert _{L^2(\Omega )}. \end{aligned}$$

This completes proof of the lemma.$\square$

### Error estimates for the semidiscrete scheme

Now we can state the error estimate for the nonsmooth initial data $v\in L^2(\Omega )$.

#### **Theorem 3.1**

Let $u$ and $u_h$ be the solutions of problem (1.1) and (3.2) with $v \in L^2(\Omega )$ and $v_h=P_h v$, respectively. Then for $t>0$, there holds:

$$\begin{aligned} \Vert u(t)-u_h(t) \Vert _{L^2(\Omega )} + h\Vert \nabla (u(t)-u_h(t)) \Vert _{L^2(\Omega )} \le ch^{2} t^{-(1-\alpha )} \Vert v\Vert _{L^2(\Omega )}. \end{aligned}$$

#### *Proof*

The error $e(t):=u(t)-u_h(t)$ can be represented as

$$\begin{aligned} e(t)=\frac{1}{2\pi \mathrm {i}}\int _{\Gamma } e^{zt} \frac{g(z)}{z} (w-w_h) \,dz, \end{aligned}$$

with $w=(g(z)I+A)^{-1}v$ and $w_h=(g(z)I+A_h)^{-1}P_h v$. By Lemma 3.4 and the argument in the proof of Theorem 2.1 we have

$$\begin{aligned} \Vert \nabla e(t) \Vert _{L^2(\Omega )}\le ch \Vert v \Vert _{L^2(\Omega )} \int _{\Gamma } e^{\mathfrak {R}(z)t} \frac{|g(z)|}{|z|} \,|dz| \le cht^{-(1-\alpha )} \Vert v \Vert _{L^2(\Omega )}. \end{aligned}$$

A similar argument also yields the $L^2(\Omega )$-estimate.$\square$

Next we turn to the case of smooth initial data, i.e., $v\in \dot{H}^{2}(\Omega )$ and $v_h\in R_hv$. We take again contour $\Gamma =\Gamma _{1/t,\pi -\theta }$. Then the error $e(t)=u(t)-u_h(t)$ can be represented as

$$\begin{aligned} e(t)=\frac{1}{2\pi \mathrm {i}}\int _{\Gamma } e^{zt} \frac{g(z)}{z} \left( (g(z)I+A)^{-1}-(g(z)I+A_h)^{-1}R_h\right) v \,dz. \end{aligned}$$

By the equality

$$\begin{aligned} \frac{g(z)}{z} (g(z)I+A)^{-1} = z^{-1}I-z^{-1}(g(z)I+A)^{-1}A, \end{aligned}$$

we can obtain

3.10 $$\begin{aligned} e(t)=\frac{1}{2\pi \mathrm {i}}\left( \int _{\Gamma } e^{zt} z^{-1} (w_h(z)-w(z)) \,dz + \int _{\Gamma } e^{zt} z^{-1} (v-R_hv) \,dz\right) , \end{aligned}$$

where $w(z)=(g(z)I+A)^{-1}A v$ and $w_h(z)=(g(z)I+A_h)^{-1}A_hR_hv$. Then we derive the following error estimate.

#### **Theorem 3.2**

Let $u$ and $u_h$ be the solutions of problem (1.1) and (3.2) with $v \in \dot{H}^{2}(\Omega )$ and $v_h=R_h v$, respectively. Then for $t>0$, there holds:

3.11 $$\begin{aligned} \Vert u(t)-u_h(t) \Vert _{L^2(\Omega )} + h\Vert \nabla (u(t)-u_h(t)) \Vert _{L^2(\Omega )} \le c h^{2} \Vert v\Vert _{\dot{H}^{2}(\Omega )}. \end{aligned}$$

#### *Proof*

Let $w(z)=(g(z)I+A)^{-1}Av$ and $w_h(z)=(g(z)I+A_h)^{-1}A_hR_hv$. Then Lemmas 3.1 and 3.4, and the identity $A_hR_h=P_h A$ give

$$\begin{aligned} \Vert w(z)-w_h(z) \Vert _{L^2(\Omega )} + h\Vert \nabla (w(z)-w_h(z)) \Vert _{L^2(\Omega )} \le c h^{2} \Vert Av \Vert _{L^2(\Omega )}. \end{aligned}$$

Now it follows from this and the representation (3.10) that

$$\begin{aligned} \Vert e(t)\Vert&\le c h^2 \Vert Av \Vert _{L^2(\Omega )}\int _{\Gamma } e^{\mathfrak {R}(z)t}| z|^{-1} \,|dz|\\&\le c h^2 \Vert Av \Vert _{L^2(\Omega )}\left( \int _{1/t}^\infty e^{-rt\cos \theta } r^{-1} \,dr + \int _{-\pi +\theta }^{\pi -\theta } e^{\cos \psi } \,d\psi \right) \\&\le c h^2 \Vert Av \Vert _{L^2(\Omega )}=ch^2 \Vert v \Vert _{\dot{H}^{2}(\Omega )}. \end{aligned}$$

Hence we obtain the $L^2(\Omega )$-error estimate. The $H^1(\Omega )$-error estimate follows analogously.

#### *Remark 3.1*

For smooth initial data $v\in \dot{H}^{2}(\Omega )$, we may also take the approximation $v_h=P_hv$. Then the error can be split into

$$\begin{aligned} e(t)=S(t)v-S_h(t)P_hv=(S(t)v-S_h(t)R_hv)+(S_h(t)R_hv-S_h(t)P_hv). \end{aligned}$$

Theorem 3.2 gives an estimate of the first term. A bound for the second term follows from Lemmas 3.1 and 3.2

$$\begin{aligned} \Vert S_h(t)(P_hv-R_hv) \Vert _{\dot{H}^{p}(\Omega )} \le c\Vert P_hv-R_hv \Vert _{\dot{H}^{p}(\Omega )} \le ch^{2-p}\Vert v\Vert _{\dot{H}^{2}(\Omega )}. \end{aligned}$$

Thus the error estimate (3.11) holds for the initial approximation $v_h=P_hv$. It follows from this, Theorem 3.1, and interpolation that for all $q\in [0,2]$ and $v_h=P_hv$, there holds

$$\begin{aligned} \Vert u(t)-u_h(t) \Vert _{L^2(\Omega )} + h\Vert \nabla (u(t)-u_h(t)) \Vert _{L^2(\Omega )} \le c h^{2} t^{-(1-\alpha )(2-q)/2} \Vert v\Vert _{\dot{H}^q (\Omega )}. \end{aligned}$$

#### *Remark 3.2*

If the initial data is very weak, i.e., $v\in \dot{H}^q(\Omega )$ with $-1<q<0$, Then the argument of [8, Theorem 2] yields the following optimal error estimate for the semidiscrete finite element approximation (1.2)

3.12 $$\begin{aligned} \Vert u(t)-u_h(t) \Vert _{L^2(\Omega )} + h\Vert \nabla (u(t)-u_h(t)) \Vert _{L^2(\Omega )} \le c h^{2+q} t^{-(1-\alpha )} \Vert v\Vert _{\dot{H}^{q}(\Omega )}.\nonumber \\ \end{aligned}$$

## Fully discrete schemes

Now we develop two fully discrete schemes for problem (1.1) based on convolution quadrature (see [4, 13–15] for detailed discussions), and derive optimal error estimates for both smooth and nonsmooth initial data.

### Convolution quadrature

First we briefly describe the abstract framework in [4, Sections 2 and 3], which is instrumental in the development and analysis of fully discrete schemes. Let $K$ be a complex valued or operator valued function that is analytic in a sector $\Sigma _{\pi -\theta }, \theta \in (0,\pi /2)$ and is bounded by

4.1 $$\begin{aligned} \Vert K(z)\Vert \le M|z|^{-\mu }\quad \forall z\in \Sigma _{\pi -\theta }, \end{aligned}$$

for some real numbers $\mu$ and $M$. Then $K(z)$ is the Laplace transform of a distribution $k$ on the real line, which vanishes for $t<0$, has its singular support empty or concentrated at $t=0$, and which is an analytic function for $t>0$. For $t>0$, the analytic function $k(t)$ is given by the inversion formula

$$\begin{aligned} k(t) = \frac{1}{2\pi \mathrm {i}}\int _\Gamma K(z)e^{zt}dz, \ \ t>0, \end{aligned}$$

where $\Gamma$ is a contour lying in the sector of analyticity, parallel to its boundary and oriented with increasing imaginary part. With $\partial _t$ being time differentiation, we define $K(\partial _t)$ as the operator of (distributional) convolution with the kernel $k:K(\partial _t)g=k*g$ for a function $g(t)$ with suitable smoothness.

A convolution quadrature approximates $K(\partial _t)g(t)$ by a discrete convolution $K(\bar{\partial }_\tau ) g(t)$. Specifically, we divide the time interval $[0,T]$ into $N$ equal subintervals with a time step size $\tau =T/N$, and define the approximation:

$$\begin{aligned} K(\bar{\partial }_\tau ) g(t) = \sum _{0\le j\tau \le t}\omega _jg(t-j\tau ), \ \ t>0, \end{aligned}$$

where the quadrature weights $\{\omega _j\}_{j=0}^\infty$ are determined by the generating function

$$\begin{aligned} \sum _{j=0}^\infty \omega _j\xi ^j = K(\delta (\xi )/\tau ). \end{aligned}$$

Here $\delta$ is the quotient of the generating polynomials of a stable and consistent linear multistep method. In this work, we consider the backward Euler (BE) method and second-order backward difference (SBD) method, for which

$$\begin{aligned} \delta (\xi ) = \left\{ \begin{array}{ll} (1-\xi ), \qquad &{}\ \ \hbox {BE},\\ (1-\xi ) + (1-\xi )^2/2, &{}\ \ \hbox {SBD}. \end{array}\right. \end{aligned}$$

Now we specialize the construction to the semidiscrete problem (3.2). By integrating (3.2) from $0$ to $t$, we arrive at a representation of the semidiscrete solution $u_h$

$$\begin{aligned} u_h + (\gamma \partial _t^{\alpha -1}+\partial _t^{-1})A_h u_h =v_h + \partial _t^{-1}f_h. \end{aligned}$$

where $\partial _t^{\beta } u, \beta <0$, denotes the Riemann–Liouville integral $\partial _t^\beta u= \frac{1}{\Gamma (-\beta )}\int _0^t(t-s)^{-\beta -1}u(s)ds$. The left-hand side is a convolution, which we approximate at $t_n=n\tau$ with $U_h^n$ by

$$\begin{aligned} U_h^n + (\gamma \bar{\partial }_\tau ^{\alpha -1} + \bar{\partial }_\tau ^{-1})A_hU_h^n = v_h + \bar{\partial }_\tau ^{-1}(t_n), \end{aligned}$$

where the symbols $\bar{\partial }_\tau ^{\alpha -1}$ and $\bar{\partial }_\tau ^{-1}$ refer to relevant convolution quadrature generated by the respective linear multistep method. For the convenience of numerical implementation, we rewrite them in a time stepping form.

#### The backward Euler (BE) method

The BE method is given by: Find $U_h^n$ for $n=1,2,\ldots ,N$ such that

4.2 $$\begin{aligned} U_h^n + \left( \gamma \bar{\partial }_\tau ^{\alpha -1} + \bar{\partial }_\tau ^{-1}\right) A_hU_h^n = v_h + \bar{\partial }_\tau ^{-1} f_h(t_n) \end{aligned}$$

with the convolution quadratures $\bar{\partial }_\tau ^{\alpha -1}$ and $\bar{\partial }_\tau ^{-1}$ generated by the BE method. By applying $\bar{\partial }_\tau$ to the scheme (4.2) and the associativity of convolution, we deduce that it can be rewritten as: with $U_h^0=v_h\in X_h$ and $F_h^n=f_h(t_n)$, find $U_h^n$ for $n=1,2,\ldots ,N$ such that

4.3 $$\begin{aligned} \tau ^{-1}\left( U_h^n-U_h^{n-1}\right) + \gamma \bar{\partial }_\tau ^\alpha (A_hU_h^n) + A_h U_h^n = F_h^n. \end{aligned}$$

##### *Remark 4.1*

In the scheme (4.3), the term at $n=0$ in $\bar{\partial }_\tau ^\alpha A_hU_h^n$ can be omitted without affecting its convergence rate [15, 27].

#### The second-order backward difference (SBD) method

Now we turn to the SBD scheme. It is known that it is only first-order accurate if $g(0)\ne 0$, e.g., for $g\equiv 1$ [13, Theorem 5.1] [4, Section 3]. The first-order convergence is numerically also observed on problem (1.1). Hence, one needs to correct the scheme, and we follow the approach proposed in [4, 15]. Using the identity

$$\begin{aligned} \left( I+\left( \partial ^{\alpha -1}_t +\partial ^{-1}_t\right) A_h\right) ^{-1}=I-\left( I+\left( \partial ^{\alpha -1}_t+\partial ^{-1}_t\right) A_h\right) ^{-1}\left( \partial ^{\alpha -1}_t+\partial ^{-1}_t\right) A_h, \end{aligned}$$

we can rewrite the semidiscrete solution $u_h$ into

$$\begin{aligned} u_h&= v_h + \left( I+\left( \gamma \partial _t^{\alpha -1}+\partial _t^{-1}\right) A_h\right) ^{-1}\\&\times \left( -\left( \gamma \partial _t^{\alpha -1} + \partial _t^{-1}\right) A_hv_h + \partial ^{-1}_tf_{h,0} + \partial _t^{-1}\tilde{f}_h\right) , \end{aligned}$$

where $f_{h,0} = f_h(0)$ and $\tilde{f}_h=f_h-f_h(0)$. This leads to the convolution quadrature

4.4 $$\begin{aligned} U_h^n&= v_h+\left( I+\left( \gamma \bar{\partial }_\tau ^{\alpha -1}+\bar{\partial }_\tau ^{-1}\right) A_h\right) ^{-1} \left( -\left( \gamma \bar{\partial }_\tau ^{\alpha }\partial _t^{-1} + \partial _t^{-1}\right) A_hv_h\right. \nonumber \\&\left. +\partial _t^{-1}f_{h,0}(t_n) + \bar{\partial }_\tau ^{-1} \tilde{f}_h(t_n)\right) . \end{aligned}$$

The purpose of keeping the operator $\partial _t^{-1}$ intact in (4.4) is to achieve a second-order accuracy, cf. Lemma 4.4 below. Letting $1_\tau =(0,3/2,1,\ldots )$, and noting the identity $1_\tau =\bar{\partial }_\tau \partial ^{-1} 1$ at grid points $t_n$, and associativity of convolution, (4.4) can be rewritten as

$$\begin{aligned}&\left( I+\left( \gamma \bar{\partial }_\tau ^{\alpha -1} +\bar{\partial }_\tau ^{-1}\right) A_h\right) \left( U_h^n - v_h\right) \\&\quad = -\left( \gamma \bar{\partial }_\tau ^{\alpha -1}+\bar{\partial }_\tau ^{-1}\right) A_h1_\tau v_h + \bar{\partial }_\tau ^{-1} 1_\tau f_{h,0}(t_n) + \bar{\partial }_\tau ^{-1} \tilde{f}_h(t_n) . \end{aligned}$$

Next by applying the operator $\bar{\partial }_\tau$, we obtain

4.5 $$\begin{aligned}&\bar{\partial }_\tau \left( U_h^n-v_h\right) + \left( \gamma \bar{\partial }_\tau ^{\alpha } + I\right) A_h\left( U_h^n-v_h\right) \nonumber \\&\quad = - \left( \gamma \bar{\partial }_\tau ^{\alpha }+I\right) A_h1_\tau v_h + 1_\tau f_{h,0}(t_n) + \tilde{f}_h(t_n). \end{aligned}$$

Thus we arrive at a time stepping scheme: with $U_h^0=v_h$, find $U_h^n$ such that

$$\begin{aligned} \tau ^{-1}{\left( 3U_h^1/2-3U_h^0/2\right) } + \gamma \tilde{\partial }_\tau ^\alpha A_hU_h^1 + A_hU_h^1+ A_hU_h^0/2 = F_h^1 + F_h^0/2, \end{aligned}$$

and for $$n\ge 2$$

$$\begin{aligned} \bar{\partial }_\tau U_h^n +\gamma \tilde{\partial }_\tau ^\alpha A_hU_h^n + A_hU_h^n = F_h^n, \end{aligned}$$

where the convolution quadrature $\tilde{\partial }_\tau ^\alpha \varphi ^n$ is given by

$$\begin{aligned} \tilde{\partial }_\tau ^\alpha \varphi ^n = \tau ^{-\alpha }\left( \sum _{j=1}^n\omega ^\alpha _{n-j}\varphi ^j + \omega ^\alpha _{n-1}\varphi ^0/2\right) , \end{aligned}$$

with the weights $\{\omega _j^\alpha \}$ generated by the SBD method.

The error analysis of the fully discrete schemes (4.3) and (4.5) for the case $f\equiv 0$ will be carried out below, following the general strategy in [4, Section 4].

### Error analysis of the backward Euler method

Upon recalling the function $g(z)$ from (2.3) and denoting

4.6 $$\begin{aligned} G(z)=\left( I+g(z)^{-1}A_h\right) ^{-1}, \end{aligned}$$

we can write the difference between $u_h(t_n)$ and $U_h^n$ as

4.7 $$\begin{aligned} U_h^n-u_h(t_n)= (G(\bar{\partial }_\tau )-G(\partial _t))v_h. \end{aligned}$$

For the error analysis, we need the following estimate [13, Theorem 5.2].

#### **Lemma 4.1**

Let $K(z)$ be analytic in $\Sigma _{\pi -\theta }$ and (4.1) hold. Then for $g(t)=ct^{\beta -1}$, the convolution quadrature based on the BE satisfies

$$\begin{aligned} \Vert (K(\partial _t)-K(\bar{\partial }_\tau ))g(t) \Vert \le \left\{ \begin{array}{l@{\quad }l} ct^{\mu -1}\tau ^\beta , &{} 0<\beta \le 1, \\ ct^{\mu +\beta -2}\tau , &{} \beta \ge 1.\end{array}\right. \end{aligned}$$

Now we can state the error estimate for nonsmooth initial data $v\in L^2(\Omega )$.

#### **Lemma 4.2**

Let $u_h$ and $U_h^n$ be the solutions of problem (3.2) and (4.3) with $v\in L^2(\Omega ), U_h^0= v_h = P_hv$ and $f\equiv 0$, respectively. Then there holds

$$\begin{aligned} \Vert u_h(t_n)-U_h^n \Vert _{L^2(\Omega )} \le c \tau t_n^{-1} \Vert v\Vert _{L^2(\Omega )}. \end{aligned}$$

#### *Proof*

By (2.2) and the identity $G(z) = g(z)(g(z)I+A_h)^{-1}$ for $z\in \Sigma _{\pi -\theta }$, there holds

$$\begin{aligned} \Vert G(z)\Vert \le c \quad \forall z\in \Sigma _{\pi -\theta }. \end{aligned}$$

Then (4.7) and Lemma 4.1 (with $\mu =0$ and $\beta =1$) give

$$\begin{aligned} \Vert U_h^n-u_h(t_n) \Vert _{L^2(\Omega )} \le c \tau t_n^{-1} \Vert v_h \Vert _{L^2(\Omega )}, \end{aligned}$$

and the desired result follows directly from the $L^2(\Omega )$ stability of $P_h$.

Next we turn to smooth initial data, i.e., $v\in \dot{H}^{2}(\Omega )$.

#### **Lemma 4.3**

Let $u_h$ and $U_h^n$ be the solutions of problem (3.2) and (4.3) with $v\in \dot{H}^{2}(\Omega ), U_h^0= v_h=R_hv$ and $f\equiv 0$, respectively. Then there holds

$$\begin{aligned} \Vert u_h(t_n)-U_h^n \Vert _{L^2(\Omega )} \le c \tau t_n^{-\alpha } \Vert Av \Vert _{L^2(\Omega )}. \end{aligned}$$

#### *Proof*

With the identity

$$\begin{aligned} A_h^{-1}(I+g(z)^{-1}A_h)^{-1}= A_h^{-1}-(g(z)I+A_h)^{-1}, \end{aligned}$$

and denoting $G_s(z)= -(g(z)I+A_h)^{-1}$, the error $U_h^n-u_h(t_n)$ can be represented by

$$\begin{aligned} U_h^n-u_h(t_n)= (G_s(\bar{\partial }_\tau )-G_s(\partial _t))A_h v_h. \end{aligned}$$

From (2.2) and Lemma 2.1 we deduce

$$\begin{aligned} \Vert G_s(z)\Vert \le M |g(z)|^{-1}=M\bigg |\frac{1+\gamma z^\alpha }{z}\bigg | \le M(|z|^{-1}+\gamma |z|^{\alpha -1})\quad \forall z\in \Sigma _{\pi -\theta }. \end{aligned}$$

Now Lemma 4.1 (with $\mu =1-\alpha$ and $\beta =1$) gives

$$\begin{aligned} \Vert U_h^n-u_h(t_n) \Vert _{L^2(\Omega )} \le c \tau t_n^{-\alpha } \Vert A_h v_h \Vert _{L^2(\Omega )}, \end{aligned}$$

and the desired estimate follows directly from the identity $A_hR_h=P_hA$.$\square$

#### *Remark 4.2*

By Lemma 4.3, the error estimate exhibits a singular behavior of order $t^{-\alpha }$ as $t\rightarrow 0^+$, even for smooth initial data $v\in \dot{H}^{2}(\Omega )$. Nonetheless, as $\alpha \rightarrow 0^+$, problem (1.1) reduces to the standard parabolic equation, and accordingly the singular behavior disappears for smooth data, which coincides with the parabolic counterpart [29].

Now we can state error estimates for the fully discrete scheme (4.3) with smooth and nonsmooth initial data, by the triangle inequality, Theorems 3.1 and 3.2, Lemmas 4.2 and 4.3, respectively for the nonsmooth and smooth initial data.

#### **Theorem 4.1**

Let $u$ and $U_h^n$ be the solutions of problem (1.1) and (4.3) with $U_h^0= v_h$ and $f\equiv 0$, respectively. Then the following estimates hold.

- If $v\in \dot{H}^{2}(\Omega )$ and $v_h=R_h v$, then
  $$\begin{aligned} \Vert u(t_n)-U_h^n \Vert _{L^2(\Omega )} \le c (\tau t_n^{-\alpha } + h^2) \Vert v \Vert _{\dot{H}^{2}(\Omega )}. \end{aligned}$$
- If $v\in L^2(\Omega )$ and $v_h=P_hv$, then
  $$\begin{aligned} \Vert u(t_n)-U_h^n \Vert _{L^2(\Omega )} \le c (\tau t_n^{-1} + h^2 t_n^{\alpha -1}) \Vert v\Vert _{L^2(\Omega )}. \end{aligned}$$

#### *Remark 4.3*

For $v\in \dot{H}^{2}(\Omega )$, we can also choose $v_h=P_hv$. Let $\overline{U}^n_h$ be the corresponding solution of the fully discrete scheme with $v_h=P_hv$. By the stability of the scheme, a direct consequence of Lemma 4.3, we have

$$\begin{aligned} \Vert U_h^n-\overline{U}^n_h\Vert _{L^2(\Omega )}\le c\Vert R_h v- P_h v \Vert _{L^2(\Omega )}\le ch^2\Vert v \Vert _{\dot{H}^{2}(\Omega )}. \end{aligned}$$

Thus the estimate in Theorem 4.1(a) still holds for $v_h=P_hv$. Then by interpolation with the estimate for $v\in L^2(\Omega )$, we deduce

$$\begin{aligned} \Vert u(t_n)-U_h^n \Vert _{L^2(\Omega )} \le c (\tau t_n^{-1+(1-\alpha )q/2} +h^{2} t_n^{-(1-\alpha )(2-q)/2}) \Vert v \Vert _{\dot{H}^q (\Omega )}, \quad 0 \le q \le 2. \end{aligned}$$

#### *Remark 4.4*

In case of very weak initial data, i.e., $v\in \dot{H}^{q}(\Omega )$ with $-1<q<0$, by Lemma 4.2, the inverse inequality [3, pp. 140] and Lemma 3.1 we have

$$\begin{aligned}&\Vert u_h(t_n) - U_h^n \Vert _{L^2(\Omega )} \le c\tau t_n^{-1} \Vert P_h v \Vert _{L^2(\Omega )}\\&\quad \le c \tau h^{q} t_n^{-1} \Vert P_h v \Vert _{\dot{H}^{q}(\Omega )} \le c \tau h^q t_n^{-1} \Vert v \Vert _{\dot{H}^{q}(\Omega )}. \end{aligned}$$

This and Remark 3.2 yield the following error estimate

$$\begin{aligned} \Vert u(t_n) - U_h^n \Vert _{L^2(\Omega )} \le c (\tau h^q t_n^{-1} + h^{2+q}t_n^{\alpha -1}) \Vert v \Vert _{\dot{H}^{q}(\Omega )}. \end{aligned}$$

### Error analysis of the second-order backward difference method

With $G(z)=-g(z)^{-1}z(I+g(z)^{-1}A_h)^{-1}A_h=-zA_h(g(z)I+A_h)^{-1}$, we have

4.8 $$\begin{aligned} u_h-U_h^n=(G(\partial _t)-G(\bar{\partial }_\tau ))\partial _t^{-1}v_h. \end{aligned}$$

Like Lemma 4.1, the following estimate holds (see [13, Theorem 5.2] [14, Theorem 2.2]).

#### **Lemma 4.4**

Let $K(z)$ be analytic in $\Sigma _{\pi -\theta }$ and (4.1) hold. Then for $g(t)=ct^{\beta -1}$, the convolution quadrature based on the SBD satisfies

$$\begin{aligned} \Vert (K(\partial _t)-K(\bar{\partial }_\tau )g(t) \Vert \le \left\{ \begin{array}{ll} ct^{\mu -1}\tau ^\beta , &{}~~0<\beta \le 2, \\ ct^{\mu +\beta -3}\tau ^2, &{}~~\beta \ge 2.\end{array}\right. \end{aligned}$$

Now we can state the error estimate for nonsmooth initial data $v\in L^2(\Omega )$.

#### **Lemma 4.5**

Let $u_h$ and $U_h^n$ be the solutions of problem (3.2) and (4.5) with $v\in L^2(\Omega ), U_h^0= v_h = P_hv$ and $f\equiv 0$, respectively. Then there holds

$$\begin{aligned} \Vert u_h(t_n)-U_h^n \Vert _{L^2(\Omega )} \le c \tau ^2 t_n^{-2} \Vert v\Vert _{L^2(\Omega )}. \end{aligned}$$

#### *Proof*

By (2.2) and the identity

$$\begin{aligned} G(z) = -z A_h(g(z)I+A_h)^{-1}=-z(I-g(z)(g(z)I+A_h)^{-1})\quad \forall z\in \Sigma _{\pi -\theta }, \end{aligned}$$

there holds

$$\begin{aligned} \Vert G(z)\Vert \le c|z|, \quad \forall z\in \Sigma _{\pi -\theta }. \end{aligned}$$

Then (4.8) and Lemma 4.4 (with $\mu =-1$ and $\beta =2$) give

$$\begin{aligned} \Vert U_h^n-u_h(t_n) \Vert _{L^2(\Omega )} \le c\tau ^2 t_n^{-2} \Vert v_h \Vert _{L^2(\Omega )}, \end{aligned}$$

and the desired result follows directly from the $L^2(\Omega )$ stability of $P_h$.$\square$

Next we turn to smooth initial data $v\in \dot{H}^{2}(\Omega )$.

#### **Lemma 4.6**

Let $u_h$ and $U_h^n$ be the solutions of problem (3.2) and (4.5) with $v\in \dot{H}^{2}(\Omega ), U_h^0= v_h=R_hv$ and $f\equiv 0$, respectively. Then there holds

$$\begin{aligned} \Vert u_h(t_n)-U_h^n \Vert _{L^2(\Omega )} \le c \tau ^2 t_n^{-1-\alpha } \Vert Av \Vert _{L^2(\Omega )}. \end{aligned}$$

#### *Proof*

By setting $G_s(z)= -z(g(z)I+A_h)^{-1}, U_h^n-u_h(t_n)$ can be represented by

$$\begin{aligned} U_h^n-u_h(t_n)= (G_s(\bar{\partial }_\tau )-G_s(\partial _t))A_h v_h. \end{aligned}$$

From (2.2) and Lemma 2.1 we deduce

$$\begin{aligned} \Vert G_s(z)\Vert \le M |z||g(z)|^{-1}\le (1+\gamma |z|^\alpha ), \quad \forall z\in \Sigma _{\pi -\theta }. \end{aligned}$$

Now Lemma 4.4 (with $\mu =-\alpha$ and $\beta =2$) gives

$$\begin{aligned} \Vert U_h^n-u_h(t_n)\Vert _{L^2(\Omega )} \le c\tau ^2 t_n^{-1-\alpha } \Vert A_h v_h \Vert _{L^2(\Omega )}, \end{aligned}$$

and the desired estimate follows from the identity $A_hR_h=P_hA$.$\square$

Then we have the following error estimates for the fully discrete scheme (4.5).

#### **Theorem 4.2**

Let $u$ and $U_h^n$ be solutions of problem (1.1) and (4.5) with $U_h^0$ and $f\equiv 0$, respectively. Then the following error estimates hold.

- If $v\in \dot{H}^{2}(\Omega )$, and $U_h^0=R_hv$, there holds
  $$\begin{aligned} \Vert u(t_n)-U_h^n \Vert _{L^2(\Omega )} \le c(\tau ^2 t_n^{-1-\alpha } + h^2) \Vert v\Vert _{\dot{H}^{2}(\Omega )}. \end{aligned}$$
- If $v\in L^2(\Omega )$, and $U_h^0=P_hv$, there holds
  $$\begin{aligned} \Vert u(t_n)-U_h^n \Vert _{L^2(\Omega )} \le c(\tau ^2 t_n^{-2} + h^2 t_n^{\alpha -1}) \Vert v\Vert _{L^2(\Omega )}. \end{aligned}$$

#### *Remark 4.5*

By the stability of the scheme, a direct consequence of Lemma 4.6, and the argument in Remark 4.3, the estimate in Theorem 4.2(a) still holds for $v_h=P_hv$. Then by interpolation we have

$$\begin{aligned} \Vert u(t_n)-U_h^n \Vert _{L^2(\Omega )} \le c(\tau ^2 t_n^{-2+(1-\alpha )q/2} +h^{2} t^{-(1-\alpha )(2-q)/2}) \Vert v\Vert _{\dot{H}^q (\Omega )}, \quad 0 \le q \le 2. \end{aligned}$$

#### *Remark 4.6*

In case of very weak initial data $v\in \dot{H}^{q}(\Omega ), -1<q<0$, the argument in Remark 4.4 yields

$$\begin{aligned} \Vert u(t_n) - U_h^n \Vert _{L^2(\Omega )} \le c(\tau ^2 h^q t_n^{-2} + h^{2+q}t^{\alpha -1}) \Vert v \Vert _{\dot{H}^{q}(\Omega )}. \end{aligned}$$

## Numerical results

In this part, we present numerical results to verify the convergence theory in Sects. 3 and 4. We shall consider one- and two-dimensional examples with smooth, nonsmooth and very weak initial data. In the one-dimensional case, we take $\Omega =(0,1)$, and in the two-dimensional case $\Omega =(0,1)^2$. Here we use the notation $\chi _S$ for the characteristic function of the set $S$. The following four cases are considered.

- smooth: $v=\sin (2\pi x)$ which is in $H^2(\Omega )\cap H_0^1(\Omega )$.
- nonsmooth: $v=\chi _{(0,1/2]}$; the jump at $x=1/2$ and $v(0) \not = 0$ lead to $v \notin \dot{H}^1(\Omega )$; but for any $\epsilon \in (0,1/2), v\in \dot{H}^{{1/2}-\epsilon }(\Omega )$.
- very weak data: $v=\delta _{1/2}(x)$ which is a Dirac $\delta$-function concentrated at $x=0.5$. By Sobolev imbedding theorem, $v\in \dot{H}^{-1/2-\epsilon }(\Omega )$ for $\epsilon >0$.
- two-dimensional example: $v=\chi _{(0,1/2]\times (0,1)}$ which is in $\dot{H}^{{1/2}-\epsilon }(\Omega )$ for any $\epsilon >(0, 1/2)$.

In our experiments, we fix the parameter $\gamma =1$ in (1.1) for all cases. We examine separately the spatial and temporal convergence rates at $t=0.1$. For the case of nonsmooth initial data, we are especially interested in the errors for $t$ close to zero. The exact solutions to these examples can be expressed in terms of generalized Mittag–Leffler functions, which however is difficult to compute, and hence we compute the reference solution on a very refined mesh. We report the normalized errors $\Vert e^n \Vert _{L^2(\Omega )}/\Vert v \Vert _{L^2(\Omega )}$ and $\Vert e^n \Vert _{\dot{H}^1(\Omega )}/\Vert v \Vert _{L^2(\Omega )}, e^n = u(t_n)-U_h^n$, for both smooth and nonsmooth data.

In our computation, we divide the unit interval $(0,1)$ into $K=2^k$ equally spaced subintervals, with a mesh size $h=1/K$. The finite element space $X_h$ consists of continuous piecewise linear functions. Similarly, we take the uniform temporal mesh with a time step size $\tau =t/N$, with $t$ being the time of interest.

### Numerical results for example (a)

**Fig. 1:**
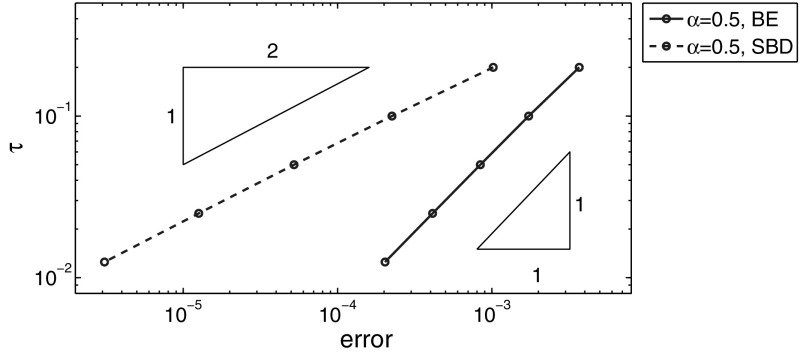
Error plots for example (a) at $t=0.1$, with $\alpha =0.5$ and $h=2^{-11}$

First, we fix the mesh size $h$ at $h = 2^{-11}$ so that the error incurred by spatial discretization is negligible, which enable us to examine the temporal convergence rate. In Table 1, we show the $L^2(\Omega )$-norm of the error at $t=0.1$ for different $\alpha$ values. In the table, BE and SBD denote the backward Euler method and the second-order backward difference method, respectively, rate refers to the empirical convergence rate when the time step size $\tau$ (or the mesh size $h$) halves, and the numbers in the bracket denote theoretical convergence rates. In Fig. 1 we plot the results for $\alpha =0.5$ in a log-log scale. A convergence rate of order $O(\tau )$ and $O(\tau ^2)$ is observed for the BE method and the SBD method, respectively, which agrees well with our convergence theory. Further, we observe that the error decreases as the fractional order $\alpha$ increases.

**Table 1:** The $L^2(\Omega )$-norm of the error for example (a): $t=0.1$ and $h=2^{-11}$

	$\tau$	$1/5$	$1/10$	$1/20$	$1/40$	$1/80$	Rate
BE	$\alpha =0.1$	6.75e$-$3	2.42e$-$3	1.00e$-$3	4.55e$-$4	2.15e$-$4	$\approx$1.15 (1.00)
	$\alpha =0.5$	3.68e$-$3	1.73e$-$3	8.42e$-$4	4.13e$-$4	2.03e$-$4	$\approx$1.04 (1.00)
	$\alpha =0.9$	4.12e$-$4	2.03e$-$4	1.00e$-$4	4.96e$-$5	2.43e$-$5	$\approx$1.03 (1.00)
SBD	$\alpha =0.1$	5.59e$-$3	4.82e$-$4	1.18e$-$4	2.77e$-$5	6.66e$-$6	$\approx$2.06 (2.00)
	$\alpha =0.5$	1.05e$-$3	2.39e$-$4	5.33e$-$5	1.28e$-$5	3.14e$-$6	$\approx$2.08 (2.00)
	$\alpha =0.9$	7.62e$-$5	1.64e$-$5	3.86e$-$6	9.48e$-$7	2.46e$-$7	$\approx$2.06 (2.00)

**Fig. 2:**
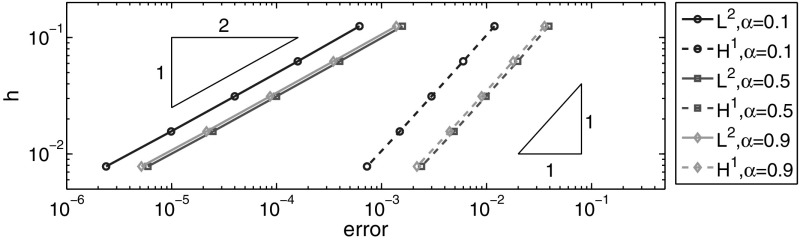
Error for example (a): $t=0.1, \tau = 2^{-5},\alpha =0.1, 0.5$ and $0.9$

In Table 2 and Fig. 2, we show the $L^2(\Omega )$- and $H^1(\Omega )$-norms of the error at $t=0.1$ for the BE scheme. We set $\tau =2\times 10^{-5}$ and check the spatial convergence rate. The numerical results show $O(h^{2})$ and $O(h)$ convergence rates respectively for the $L^2(\Omega )$- and $H^1(\Omega )$-norms of the error, which fully confirm Theorem 3.2. Further, the empirical convergence rate is almost independent of the fractional order $\alpha$.

**Table 2:** Error for example (a): $t=0.1, h = 2^{-k}$ and $\tau =5\times 10^{-5}$

$\alpha$	$k$	$3$	$4$	$5$	$6$	$7$	Rate
$\alpha =0.1$	$L^2$-norm	6.16e$-$4	1.59e$-$4	4.00e$-$5	9.90e$-$6	2.38e$-$6	$\approx$2.01 (2.00)
	$H^1$-norm	1.19e$-$2	5.99e$-$3	2.99e$-$3	1.49e$-$3	7.26e$-$4	$\approx$1.01 (1.00)
$\alpha =0.5$	$L^2$-norm	1.58e$-$3	4.00e$-$4	1.00e$-$4	2.48e$-$5	5.95e$-$6	$\approx$2.01 (2.00)
	$H^1$-norm	3.92e$-$2	1.98e$-$2	9.88e$-$3	4.91e$-$3	2.40e$-$3	$\approx$1.01 (1.00)
$\alpha =0.9$	$L^2$-norm	1.38e$-$3	3.47e$-$4	8.67e$-$5	2.15e$-$5	5.16e$-$6	$\approx$2.01 (2.00)
	$H^1$-norm	3.56e$-$2	1.79e$-$2	8.96e$-$3	4.45e$-$3	2.17e$-$3	$\approx$ 1.01 (1.00)

### Numerical results for example (b)

In Tables 3 and 4 we present the results for example (b). The temporal convergence rate is $O(\tau )$ and $O(\tau ^2)$ for the BE and the SBD method, respectively, cf. Table 3, and the spatial convergence rate is of order $O(h^2)$ in $L^2(\Omega )$-norm and $O(h)$ in $H^1(\Omega )$-norm, cf. Table 4. For nonsmooth initial data, we are especially interested in errors for $t$ close to zero. Thus we also present the error at $t = 0.01$ and $t = 0.001$ in Table 4. The numerical results fully confirm the predicted rates.

**Fig. 3:**
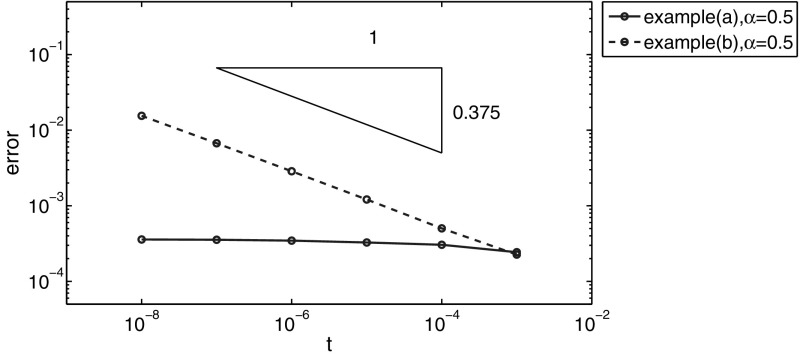
Error plots for examples (a) and (b) with $h=2^{-6}, \alpha =0.5$ for $t\rightarrow 0$

Further, in Table 5 and Fig. 3 we show the $L^2(\Omega )$-norm of the error for examples (a) and (b), for fixed $h = 2^{-6}$ and $t\rightarrow 0$. To check the spatial discretization error, we fix time step $\tau$ at $\tau =t/1{,}000$ and use the SBD method so that the temporal discretization error is negligible. We observe that in the smooth case, i.e., example (a), the spatial error essentially stays unchanged, whereas in the nonsmooth case, i.e., example (b), it deteriorates as $t\rightarrow 0$. In example (b) the initial data $v\in \dot{H}^{1/2-\epsilon }(\Omega )$ for any $\epsilon >0$, and by Remark 4.5, the error grows like $O(t^{-3\alpha /4})$ as $t\rightarrow 0$. The empirical rate in Table 5 and Fig. 3 agrees well with the theoretical prediction, i.e., $-3\alpha /4=-0.375$ for $\alpha =0.5$.

**Table 3:** The $L^2(\Omega )$-norm of the error for example (b) at $t=0.1$, with $h=2^{-11}$

	$\tau$	$1/5$	$1/10$	$1/20$	$1/40$	$1/80$	Rate
BE	$\alpha =0.1$	2.82e$-$2	1.42e$-$2	7.13e$-$3	3.56e$-$3	1.76e$-$3	$\approx$1.00 (1.00)
	$\alpha =0.5$	8.67e$-$3	4.18e$-$3	2.05e$-$3	1.01e$-$3	4.97e$-$4	$\approx$1.02 (1.00)
	$\alpha =0.9$	9.06e$-$4	4.47e$-$4	2.21e$-$4	1.09e$-$4	5.42e$-$5	$\approx$1.02 (1.00)
SBD	$\alpha =0.1$	7.14e$-$3	1.61e$-$3	3.92e$-$4	9.63e$-$5	2.38e$-$5	$\approx$2.05 (2.00)
	$\alpha =0.5$	2.46e$-$3	5.05e$-$4	1.17e$-$4	2.82e$-$5	6.91e$-$6	$\approx$2.06 (2.00)
	$\alpha =0.9$	1.67e$-$4	3.58e$-$5	8.40e$-$6	2.04e$-$6	5.11e$-$7	$\approx$2.08 (2.00)

**Table 4:** Error for example (b): $\alpha =0.5,h = 2^{-k}$ and $N=1{,}000$

$t$	$k$	$3$	$4$	$5$	$6$	$7$	Rate
$t=0.1$	$L^2$-norm	1.63e$-$3	4.09e$-$4	1.02e$-$4	2.55e$-$5	6.30e$-$6	$\approx$2.00 (2.00)
	$H^1$-norm	4.04e$-$2	2.02e$-$2	1.01e$-$2	5.04e$-$3	2.51e$-$3	$\approx$1.00 (1.00)
$t=0.01$	$L^2$-norm	5.87e$-$3	1.47e$-$3	3.66e$-$4	9.13e$-$5	2.26e$-$5	$\approx$2.00 (2.00)
	$H^1$-norm	1.62e$-$1	8.08e$-$2	4.04e$-$2	2.02e$-$2	1.00e$-$2	$\approx$1.00 (1.00)
$t=0.001$	$L^2$-norm	1.47e$-$2	3.66e$-$3	9.15e$-$4	2.28e$-$4	5.65e$-$5	$\approx$2.00 (2.00)
	$H^1$-norm	4.48e$-$1	2.24e$-$1	1.12e$-$1	5.60e$-$2	2.78e$-$2	$\approx$1.00 (1.00)

**Table 5:** The $L^2(\Omega )$-norm of the error for examples (a) and (b) with $\alpha =0.5, h=2^{-6}$, and $t \rightarrow 0$

$t$	1e$-$3	1e$-$4	1e$-$5	1e$-$6	1e$-$7	1e$-$8	Rate
(a)	2.48e$-$4	3.07e$-$4	3.27e$-$4	3.46e$-$4	3.55e$-$4	3.58e$-$4	$\approx -$0.02 (0)
(b)	2.28e$-$4	5.07e$-$4	1.22e$-$3	2.89e$-$3	6.78e$-$3	1.56e$-$2	$\approx -$0.37 ($-$0.37)

### Numerical results for example (c)

In the case of very weak data, according to Remarks 4.4 and 4.6, we can only expect spatial convergence for a small time step size $\tau$. The results in Table 6 indicate a superconvergence phenomenon with a rate $O(h^2)$ in the $L^2(\Omega )$-norm and $O(h)$ in the $H^1(\Omega )$-norm. This is attributed to the fact that in one dimension the solution with the Dirac $\delta$-function as the initial data is smooth from both sides of the support point and the finite element spaces $X_h$ have good approximation property. When the singularity point $x=1/2$ is not aligned with the grid, Table 7 shows an $O(h^{3/2})$ and $O(h^{1/2})$ rate for the $L^2(\Omega )$- and $H^1(\Omega )$-norm of the error, respectively.

**Table 6:** Error for example (c): $\alpha =0.5,h = 2^{-k}$, and $N=1{,}000$

$t$	$k$	$3$	$4$	$5$	$6$	$7$	Rate
$t=0.1$	$L^2$-norm	1.19e$-$4	2.98e$-$5	7.45e$-$6	1.86e$-$6	4.62e$-$7	$\approx$2.00 (1.50)
	$H^1$-norm	5.35e$-$3	2.69e$-$3	1.35e$-$3	6.72e$-$4	3.34e$-$4	$\approx$1.00 (0.50)
$t=0.01$	$L^2$-norm	2.41e$-$3	6.04e$-$4	1.51e$-$4	3.77e$-$5	9.31e$-$6	$\approx$2.00 (1.50)
	$H^1$-norm	3.98e$-$2	1.99e$-$2	9.92e$-$3	4.95e$-$3	2.46e$-$3	$\approx$1.00 (0.50)
$t=0.001$	$L^2$-norm	1.25e$-$2	3.12e$-$3	7.80e$-$4	1.94e$-$4	4.83e$-$5	$\approx$2.00 (1.50)
	$H^1$-norm	5.00e$-$1	2.50e$-$1	1.25e$-$1	6.23e$-$2	3.09e$-$2	$\approx$1.00 (0.50)

**Table 7:** Error for example (c): $\alpha =0.5,h = 1/(2^{k}+1)$ and $N=1{,}000$

$t$	$k$	$3$	$4$	$5$	$6$	$7$	Rate
$t=0.1$	$L^2$-norm	5.84e$-$3	2.22e$-$3	8.15e$-$4	2.93e$-$4	1.04e$-$4	$\approx$1.50 (1.50)
	$H^1$-norm	1.79e$-$1	1.29e$-$1	9.16e$-$2	6.44e$-$2	4.45e$-$2	$\approx$0.52 (0.50)
$t=0.01$	$L^2$-norm	2.42e$-$2	9.54e$-$3	3.57e$-$3	1.30e$-$3	4.63e$-$4	$\approx$1.48 (1.50)
	$H^1$-norm	7.77e$-$1	5.68e$-$1	4.07e$-$1	2.87e$-$1	1.98e$-$1	$\approx$0.51 (0.50)
$t=0.001$	$L^2$-norm	8.01e$-$2	3.27e$-$2	1.25e$-$2	4.57e$-$3	1.64e$-$3	$\approx$1.46 (1.50)
	$H^1$-norm	2.65e0	1.97e0	1.43e0	1.02e0	7.05e$-$1	$\approx$0.49 (0.50)

### Numerical results for example (d)

**Fig. 4:**
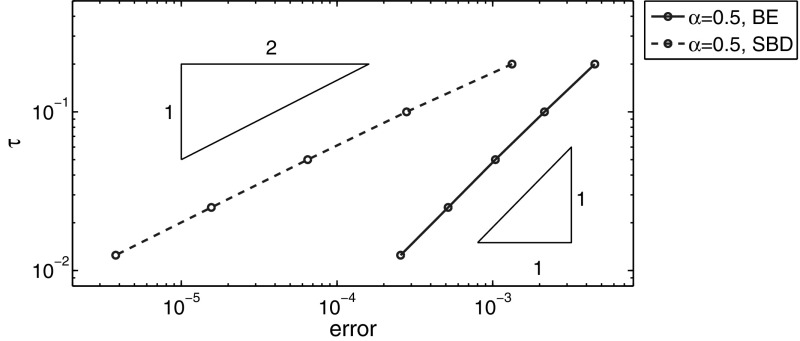
Error plots for example (d) at $t=0.1$ with $\alpha =0.5$ and $h=2^{-9}$

**Fig. 5:**
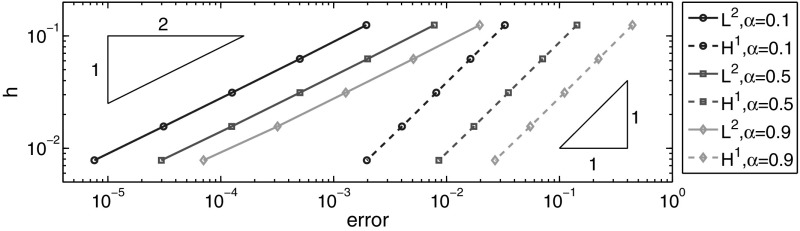
Error plots of example (d): $\alpha =0.1, 0.5, 0.9$ and $N=1{,}000$ at $t=0.1, 0.01$ and $0.001$

Here we consider a two-dimensional example on the unit square $\Omega =(0,1)^2$ for the nonsmooth initial data. To discretize the problem, we divide the unit interval $(0,1)$ into $K=2^k$ equally spaced subintervals with a mesh size $h=1/K$ so that the domain is divided into $K^2$ small squares. We get a symmetric triangulation of the domain by connecting the diagonal of each small square. Table 8 shows a temporal convergence rate of first order and second order for the BE and SBD method, respectively. Spatial errors at $t=0.1, 0.01$ and $0.001$ are showed in Table 9, which imply a convergence with a rate of $O(h^2)$ in the $L^2(\Omega )$-norm and $O(h)$ in the $H^1(\Omega )$-norm. In Figs. 4 and 5 we plot the results shown in Tables 8 and 9, respectively. All numerical results confirm our convergence theory.

**Table 8:** The $L^2$-norm of the error for example (d) at $t=0.1$, with $\alpha =0.5$ and $h=2^{-9}$

	$\tau$	$1/5$	$1/10$	$1/20$	$1/40$	$1/80$	Rate
BE	$\alpha =0.5$	4.53e$-$3	2.15e$-$3	1.04e$-$3	5.17e$-$4	2.56e$-$4	$\approx$1.03 (1.00)
SBD	$\alpha =0.5$	1.33e$-$3	2.80e$-$4	6.48e$-$5	1.56e$-$5	3.79e$-$6	$\approx$2.11 (2.00)

**Table 9:** Error for example (d): $\alpha =0.5, h = 2^{-k}$ and $N=1{,}000$

$t$	$k$	$3$	$4$	$5$	$6$	$7$	Rate
$t=0.1$	$L^2$-norm	1.95e$-$3	5.02e$-$4	1.26e$-$4	3.12e$-$5	7.61e$-$6	$\approx$2.01 (2.00)
	$H^1$-norm	3.29e$-$2	1.63e$-$2	8.11e$-$3	4.03e$-$3	1.97e$-$3	$\approx$1.00 (1.00)
$t=0.01$	$L^2$-norm	7.79e$-$3	2.00e$-$3	5.03e$-$4	1.25e$-$4	2.98e$-$5	$\approx$2.02 (2.00)
	$H^1$-norm	1.43e$-$1	7.09e$-$2	3.53e$-$2	1.75e$-$2	8.56e$-$3	$\approx$1.01 (1.00)
$t=0.001$	$L^2$-norm	1.97e$-$2	5.09e$-$3	1.28e$-$3	3.19e$-$4	7.05e$-$5	$\approx$2.00 (2.00)
	$H^1$-norm	4.44e$-$1	2.22e$-$1	1.11e$-$1	5.52e$-$2	2.69e$-$2	$\approx$1.01 (1.00)

## Concluding remarks

In this work, we have studied the homogeneous problem for the Rayleigh–Stokes equation in a second grade generalized flow. The Sobolev regularity of the solution was established using an operator theoretic approach. A space semidiscrete scheme based on the Galerkin finite element method and two fully discrete schemes based on the backward Euler method and second-order backward difference method and related convolution quadrature were developed and optimal with respect to the data regularity error estimates were provided for both semidiscrete and fully discrete schemes. Extensive numerical experiments fully confirm the sharpness of our convergence analysis.
